# An annotated checklist of the planthoppers of Iran (Hemiptera, Auchenorrhyncha, Fulgoromorpha) with distribution data

**DOI:** 10.3897/zookeys.145.1846

**Published:** 2011-11-04

**Authors:** Fariba Mozaffarian, Michael R Wilson

**Affiliations:** 1 Insect Taxonomy Research Department, Iranian Research Institute of Plant Protection, Tehran, 19395, P.O. Box 1454, Iran; 2 Department of Biodiversity & Systematic Biology, National Museum of Wales, Cardiff, CF10 3NP, UK

**Keywords:** Hemiptera, Auchenorrhyncha, Fulgoromorpha, annotated checklist, Iran

## Abstract

A list of Hemiptera Fulgoromorpha (planthoppers) of Iran is provided, based primarily on literature records from 1902 to the present. In total 15 families and 235 species are recorded, with taxonomic details. Distribution data in Iran are given. *Iranissus ephedrinus* Dlabola, 1980 is transferred from Issidae to Nogodinidae. To resolve nomenclatural difficulty the following new combinations in Issidae are given: *Iranodus dumetorus* (Dlabola, 1981), *Iranodus khatunus* (Dlabola, 1981) and *Iranodus repandus* (Dlabola, 1981). Due to published generic synonomy the following are new combinations: *Duilius seticulosus* (Lethierry, 1874), *Duilius tamaricis* (Puton & Lethierry, 1887), *Duilius tamaricicola* (Dubovsky, 1966) and *Duilius v-atrum* (Dlabola, 1985).

## Introduction

The infraorder Fulgoromorpha, also known as planthoppers, belong to the suborder Auchenorrhyncha of the order Hemiptera. Planthoppers are phytophagous and feed from the sap of a wide range of plants and in many habitats. They may occur in relatively large numbers in some habitats and under certain conditions.

The wide-ranging history of entomology in Iran has been well-documented by chapters in Abivardi (2001). The earliest available records of Fulgoromorpha in Iran were by Melichar (1902a, b) who described some species from Iran. Afshar (1937) was the first Iranian entomologist who recorded *Ommatissus binotatus lybicus* (Tropiduchidae) in the list of agricultural pests for the country. Subsequent taxonomic studies in the Persian literature mainly focused on the insect pests in both agricultural and forest ecosystems (Gharib 1966, 1998; Kheyri 1989; Ahmadi et al. 1986; Karimzadeh Esfahani et al. 1998 etc). Field expeditions were made to various parts of Iran during the 1970s by Czech entomologists, including Jiri Dlabola, along with entomologists from the Iranian Research Institute of Plant Protection. This resulted in a number of new discoveries of Iranian planthoppers, which were compiled in publications such as Dlabola (1974a, b, 1977a, b, 1980a, 1981, 1982a, b, 1983a, b, 1985 etc). Dlabola is responsible for the description of almost 50% of the currently known planthopper fauna of Iran.

Fifteen planthopper (Fulgoromorpha) families have been recorded from Iran to date. Table 1 gives details of the numbers of species in each family and the number (and percentage) known only from Iran at present. Large numbers of species have been recorded in the Cixiidae, the Delphacidae, the Flatidae and the Issidae. The fauna is characterized by high endemism in groups such as Issidae, which are flightless and have relatively limited dispersal and a narrow distribution. However, the planthopper fauna may also well be under-recorded, for instance around 100 species of Delphacidae are known from Greece (e.g. Drosopoulos et al. 1983) but only 34 so far recorded in Iran. Dlabola (1981) refers to the biotic zones in which species are found.

In the current study a list of Fulgoromorpha species recorded from Iran has been compiled, which is based on literature records and the addition of some new taxonomic data. Much basic taxonomic work is needed in the region and it is hoped that this checklist will facilitate further studies on Fulgoromorpha of Iran. To resolve nomenclatural difficulty the following new combinations in Issidae are given: *Iranodus dumetorus* (Dlabola, 1981), *Iranodus khatunus* (Dlabola, 1981) and *Iranodus repandus* (Dlabola, 1981). Due to published generic synonomy the following are new combinations: *Duilius seticulosus* (Lethierry, 1874), *Duilius tamaricis* (Puton & Lethierry, 1887), *Duilius tamaricicola* (Dubovsky, 1966) *Duilius v-atrum* (Dlabola, 1985).

**Table 1.** Numbers of species in each Fulgoromorpha family.

Families	No. of species	No. species/ % endemic
Caliscelidae	14	7 (50)
Cixiidae	51	19 (36)
Delphacidae	34	4 (11)
Derbidae	4	3 (75)
Dictyopharidae	19	10 (52)
Flatidae	30	19 (63)
Fulgoridae	3	0 (0)
Issidae	45	38 (84)
Kinnaridae	4	3 (75)
Lophopidae	1	0
Meenoplidae	3	1 (33)
Nogodinidae	5	5 (100)
Ricaniidae	3	1 (33)
Tettigometridae	14	1 (25)
Tropiduchidae	5	3 (60)
Totals	235	114 (48%)

## Economic importance of planthoppers in Iran

Planthoppers are of high economic importance mainly because they reduce crop yield through feeding and oviposition and they can transfer phytoplasmas and viruses to a wide range of plants. The planthoppers of economic importance in the United States have been reviewed by Wilson and O'Brien (1987) who recorded 150 species of planthoppers from 99 economic plants. At least 8 species in that list have been also recorded from Iran, on agricultural products such as date palm, potato, tomato, wheat, corn, rice (Gharib 1966, 1998; Haghshenas and Khajehali 2000; Khajehali et al. 2001; Nematollahi and Khajehali 2000). A further nine species have also been recorded in Iran by Abaii (2000) as pests of forest trees.

## Annotated species checklist

**Figure 1.**
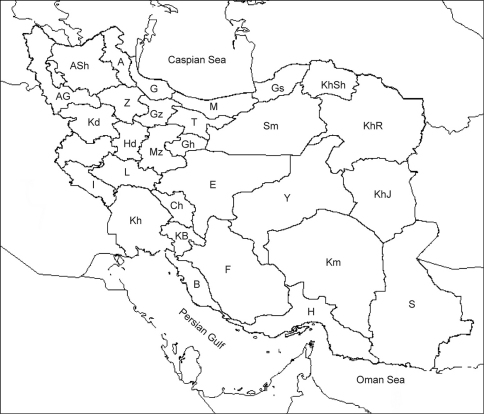
Location of the provinces of Iran (See list of the provinces for codes) **A** Ardabil **AG** Āzarbāiejan-e Gharbi **ASh** Āzarbāiejan-e Sharghi **B** Bushehr **Ch** Chahārmahāl–Bakhtiāri **E** Esfahān **F** Fārs **G** Gilān **Gh** Ghom **Gs** Golestān **Gz** Ghazvin **H** Hormozgān **Hd** Hamadān **I** Ilām **KB** Kohgiluyeh–Boyerahmad **Kd** Kordestān **Kh** Khuzestān **KhJ** Khorāsān-e Jonubi **KhR** Khorāsān-e Razavi **KhSh** Khorāsān-e Shomāli **Km** Kermān **L** Lorestān **M** Māzandarān **Mz** Markazi **S** Sistān–Baluchestān **Sm** Semnān T Tehrān **Y** Yazd **Z** Zanjān.

**Figure 2.**
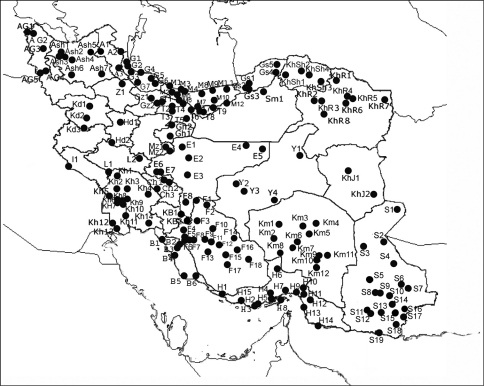
Locality map of Fulgoromorpha recorded from Iran (See List of localities for codes)

The following list is intended as an annotated checklist and not a catalogue. Emphasis is given to citations where Iran is recorded. Synonyms added after the current scientific name contain the original name, next the current name followed by its authority and next those synonyms which have been used for records from Iran. Selected taxonomic papers are cited where needed for clarity. Taxa are given in alphabetical order. Where species have only been found in Iran no further details of a wider distribution are given. Species listed in references, where no further information is given beyond the name, are cited as ‘listed'. Locality records are listed for each species. Furthermore a list of locality names including latitude, longitude, codes for the localities and a distribution map are also provided. The codes consist of two parts: an alphabetical one for the name of provinces and a numerical one for the locality (Figs 1, 2).

The extralimital distribution in the Palaearctic region was determined using various publications, including: Asche (1982), Asche and Wilson (1989), Demir and Demirsoy (2009), Demir (2007), Demir (2008), Dlabola (1965b, c, 1972, 1974b, 1977c, 1979d, 1980b, c, 1994), Dlabola and Jankovic (1981), Gnezdilov (2002), Gnezdilov and Wilson (2006), Holzinger et al. (2003), Holzinger (2007), Nast (1972), Novikov et al. (2006), Vilbaste (1980) and Wilson (2010a, b). The United Nations geoscheme was used for recording extralimital distributions in other regions and subregions of the world.

**Family Caliscelidae**

The following species were recorded in the family Issidae, subfamily Caliscelinae in early reports from Iran. This subfamily has however, been more recently given full family status (Tishechkin 1998; Emeljanov 1999; Gnezdilov 2003).

***Adenissus baluchestanicus* Dlabola, 1980**

*Adenissus baluchestanicus* Dlabola, 1980b: 184; Gnezdilov and Wilson (2006) [listed].

Iran localities: Tang-e Sarheh.

***Adenissus isinus* Dlabola, 1980**

*Adenissus isinus* Dlabola, 1980b: 185; Gnezdilov and Wilson (2006) [listed].

Iran localities: Isin.

***Adenissus zabolicus* Dlabola, 1980**

*Adenissus zabolicus* Dlabola, 1980b: 181; Gnezdilov and Wilson (2006) [listed].

Iran localities: Nehbandān.

***Adenissus zahedanicus* Dlabola, 1980**

*Adenissus zahedanicus* Dlabola, 1980b: 182; Gnezdilov and Wilson (2006) [listed].

Iran localities: Zāhedān.

***Aphelonema brunneolutea*Dlabola 1994**

*Aphelonema brunneolutea* Dlabola 1994: 57

Iran localities: Ziārān.

***Ahomocnemia chivensis* (Kusnezov, 1929)**

*Caliscelis chivensis* Kusnezov, 1929: 330

*Ahomocnemia chivensis*; Dlabola (1982a).

Iran localities: Jiroft.

Extralimital distribution: Central Asia.

***Aphelonema registanica* (Dlabola, 1961)**

*Peltonotellus registanicus* Dlabola, 1961a: 262

*Aphelonema registanica*;Dlabola (1981).

Iran localities: Kandovān (Māzandarān), Saādatshahr, Tochāl Mountain.

Extralimital distribution: Central Asia.

***Chirodisca astyages* (Dlabola, 1982)**

*Caliscelis astyages* Dlabola, 1982a: 114; Mirzayans (1995).

*Caliscelis dimidiata* not Costa, 1863; Dlabola (1981) mis-identification.

*Chirodisca astyages*; comb. n. in Emeljanov (1996a).

Iran localities: Ahvāz, Bishāpur, Borāzjān, Dārbahāre, Fasā, Ferdows-e Esfandaghe, Rafsanjān, Shirāz, Sufiān, Zeidān.

Extralimital distribution: Western Asia.

***Ommatidiotus dissimilis* (Fallén, 1806)**

*Issus dissimilis* Fallén, 1806: 123

*Ommatidiotus falléni* Stål 1863: 251; Melichar (1902a); syn. by Dlabola (1987).

*Omnatidiotus (sic) dissimilis* (Fallén, 1806); Dlabola (1981).

Iran localities: Anārak- Temin, Dasht.

Extralimital distribution: Europe, Turkey, Central and Northern Asia.

***Ommatidiotus iranicus* Horváth, 1905**

*Ommatidiotus iranicus* Horváth, 1905: 380; Nast (1972) [listed].

Iran localities: Unknown locality.

Extralimital distribution: Turkey (Demir 2008).

***Perissana bispinata* (Dlabola, 1980)**

*Anissus bispinatus* Dlabola 1980b: 179

*Perissana bispinata*; Gnezdilov and Wilson (2006).

Iran localities: Kāzerun, Kushk, Masiri, Saādatshahr.

Extralimital distribution: Iraq (Gnezdilov and Wilson 2006).

***Perissana dlabolai* Gnezdilov & Wilson, 2006**

*Adenissus circularis* Dlabola, 1980b: 186 (Preoccupied).

*Perissana dlabolai* Gnezdilov & Wilson, 2006: 15 (Replacement name for *Adenissus circularis*).

Iran localities: Bānuchārehar, Bazmān, Dehbakri, Doborji, Estahbān, Fasā, Miānjangal.

***Perissana jakowleffi* (Puton, 1890)**

*Issus jakowleffi* Puton, 1890; 233 Nast (1972) [listed].

*Perissana jakowleffi*; Gnezdilov and Wilson (2006).

Iran localities: Shāhrud.

Extralimital distribution: Azerbaijan.

***Reinhardema pasagarda* (Dlabola, 1982)**

*Homocnemia pasagarda* Dlabola, 1982a: 116

*Reinhardema pasagarda*; Gnezdilov (2010a).

Iran localities: Shul.

**Family Cixiidae**

List of subfamilies, genera, and subgenera is according to Holzinger et al. (2002).

**Subfamily Cixiinae**

**Tribe Duilini**

New combinations given are due to generic synonymy given by Holzinger et al. (2002).

***Duilius fasciata* (Horváth, 1894), comb. n.**

*Hemitropis fasciata* Horváth, 1894: 183

*Bitropis fasciata*; Dlabola (1985).

Iran localities: Ghāsemābād, Kermānshāh (Yazd province), Nikshahr.

Extralimital distribution: Transcaucasus, Turkey, Afghanistan, central Asia.

***Duilius seticulosus* (Lethierry, 1874)comb. n.**

*Haplacha seticulosa* Lethierry, 1874: 444

*Hemitropis seticulosus*; Barkhordari et al*.* (1981) [listed].

Iran localities: Unknown locality.

Extralimital distribution: Saudi Arabia (Dlabola 1979d).

***Duilius tamaricis* (Puton & Lethierry, 1887) n. comb.**

*Haplacha tamaricis* Puton & Lethierry, 1887: 309

*Hemitropis tamaricis*; Dlabola (1981).

Iran localities: Marand, Robāt-e Tork.

Extralimital distribution: Turkey, Central Asia.

***Duilius tamaricicola* (Dubovsky, 1966), comb. n.**

*Hemitropis tamaricicola* Dubovsky, 1966: 35; Dlabola (1985).

Iran localities: Borāzjān, Dārbahāre, Ghāsemābād, Kahkom Kahurak, Kermānshāh (Yazd province), Shushtar.

Extralimital distribution: Uzbekistan.

***Duilius v-atrum* (Dlabola, 1985), comb. n.**

*Hemitropis v-atrum* Dlabola, 1985: 124; Mirzayans (1995).

Iran localities: Ahram, Bāghak, Bāhukalāt, Bilāi, Geno, Ghāsemābād, Kahkom, Kahnuj, Komehr, Miānjangal, Nikshahr, Tis, Zeidān.

**Tribe Cixiini**

***Cixius (Ceratocixius) adornatus iranicus* Dlabola, 1979**

*Cixius (Ceratocixius) adornatus iranicus* Dlabola, 1979b: 233; Mirzayans (1995).

Iran localities: Hashtpar, Khalkhāl, Nāhārkhorān, Rezvāndeh, Sheikh-e Mahalleh, Zirāb.

***Cixius (Ceratocixius) cunicularius* (Linnaeus, 1767)**

*Cicada cunicularius* Linnaeus, 1767: 711

*Cixius cunicularius*; Mirzayans (1995).

Iran localities: Parehsar.

Extralimital distribution: North Africa, Europe, Central Asia, Northern and Eastern Asia.

***Cixius (Ceratocixius) pallipes* Fieber, 1876**

*Cixius (Ceratocixius) pallipes* Fieber, 1876: 191; Dlabola (1981), Mirzayans (1995).

Iran localities: Bandar Anzali, Bidruyeh, Dasht, Ghom, Hafttappeh, Hashtpar, Isin, Karaj, Evin, Lāhijān, Mollāsāni, Parehsar, Rek, Sanandaj.

Extralimital distribution: Southern, Western and Eastern Europe, North parts of Western Asia, Afghanistan.

Comments (M. Asche pers comm.): The occurrence of *pallipes* in Middle East is questionable. The true *pallipes* (described from Italy) is apparently geographically confined to the Central and West Mediterranean. In the East Mediterranean and Middle East there occurs a species described by China (1942) as *Cixius (Ceratocixius) wagneri*. This means that all geographical records of both species need confirmation.

***Cixius persicus* Distant, 1907**

*Cixius persicus* Distant, 1907: 284; Nast (1972) [listed], Dlabola (1981) [listed].

*Cixius longipennis* Melichar, 1902a: 86 (primary homonym); Melichar (1902a).

Iran localities: Nehbandān.

***Cixius (Ceratocixius) rufus* Logvinenko, 1969**

*Cixius (Ceratocixius) rufus* Logvinenko, 1969: 53; Dlabola (1985).

Iran localities: Asālem, Behshahr, Nāhārkhorān, Sabzevār.

Extralimital distribution: ‘S. Russia' (Nast, 1972).

***Cixius (Ceratocixius) simplex* (Herrich-Schäffer, 1835)**

*Flata simplex* Herrich-Schäffer, 1835: 64

*Cixius (Ceratocixius) simplex*; Dlabola (1985).

Iran localities: Pol-e Tang.

Extralimital distribution: North Africa, Europe.

***Cixius (Acanthocixius) stigmaticus* (Germar, 1818)**

*Flata stigmaticus* Germar, 1818: 199

*Cixius (Sciocixius) stigmaticus*; Dlabola (1985).

Iran localities: Kandovān (Māzandarān).

Extralimital distribution: Southern, Western and Eastern Europe.

***Tachycixius desertorum* (Fieber, 1876)**

*Cixius desertorum* Fieber, 1876: 182; (Dlabola (1981, 1985).

*Tachycixius desertorum*; comb. n. in Wagner (1939).

Iran localities: Sufiān, Eslāmābād, Ziārān.

Extralimital distribution: Southern and Eastern Europe, Turkey, Israel, Central Asia

Comments: (M. Asche pers comm.). The records of T. *desertorum* from Iran need confirmation. This species is part of a group of apparently closely related species.

**Tribe Oecleini**

***Myndus genocolus* Dlabola, 1985**

*Myndus genocolus* Dlabola, 1985: 97; Mirzayans (1995).

Iran localities: Bandar Khamir, Bandar Lengeh, Geno, Isin, Kangān.

***Myndus musivus* (Germar, 1825)**

*Flata musivus* Germar, 1825: 21; Dlabola (1981), Mirzayans (1995).

Iran localities: Orumieh, Orumieh lake.

Extralimital distribution: Europe, Central Asia.

***Myndus sarbazus* Dlabola, 1989**

*Myndus sarbazus* Dlabola, 1989: 33

Iran localities: Sarbāz.

**Tribe Pentastirini**

***Anoculiarus ornatus* Dlabola, 1985**

*Anoculiarus ornatus* Dlabola, 1985: 123

Iran localities: Bānuchārehar.

***Eumecurus apunctatus* Dlabola, 1985**

*Eumecurus apunctatus* Dlabola, 1985: 107

Iran localities: Bānuchārehar, Jiroft, Zāboli.

***Eumecurus baluchestanicus* Dlabola, 1985**

*Eumecurus baluchestanicus* Dlabola, 1985: 110

Iran localities: Masiri.

***Eumecurus (Pseumecurus) frontalis* (Melichar, 1904)**

*Oliarus frontalis* Melichar, 1904: 31

*Eumecurus (Pseumecurus) frontalis*; comb. n. in Dlabola (1985).

Iran localities: Isin.

Extralimital distribution: Northern Africa.

***Eumecurus kabulus* (Dlabola, 1957)**

*Oliarus kabulus* Dlabola, 1957a: 267

*Eumecurus kabulus*; comb. n. in Dlabola (1985).

Iran localities: Geno.

Extralimital distribution: Afghanistan.

***Eumecurus octopus* Dlabola, 1985**

*Eumecurus octopus* Dlabola, 1985: 106

Iran localities: Tang-e Sarheh.

***Eumecurus superstylus* Dlabola, 1985**

*Eumecurus superstylus* Dlabola, 1985: 108

Iran localities: Jiroft.

***Eumecurus transpunctatus* Dlabola, 1985**

*Eumecurus transpunctatus* Dlabola, 1985: 103

Iran localities: Sarbāz, Sekand.

***Eumecurus vilbastei* Dlabola, 1985**

*Eumecurus vilbastei* Dlabola, 1985: 109

Iran localities: Isin.

***Hyalesthes luteipes* Fieber, 1876**

*Hyalesthes luteipes* Fieber, 1876: 197; (Dlabola (1981, 1994), Mirzayans (1995)

Iran localities: Āb Āsk, Evin, Sirik, Tochāl Mountain.

Extralimital distribution: North Africa, Southern, Western and Eastern Europe, Turkey, Israel, Central Asia.

***Hyalesthes mlokosiewiczi* Signoret, 1879**

*Hyalesthes mlokosiewiczi* Signoret, 1879: 116; Farahbakhsh (1961) [listed], Nast (1972) [listed], Dlabola (1981), Hoch and Remane (1985) [listed], Behdad (1988), Mirzayans (1995), Emeljanov (1996b), Abaii (2000).

Iran localities: Āb Āsk, Āmol, Bidhend, Eskandari, Evin, Firuzkuh, Ghezel-Bolāgh, Gorgān, Karaj, Khorramābād, Malārd, Marāgheh, Marand, Robāt-e Tork, Rudbārak, Shāhrud, Shirāz, Tabriz, Tochāl Mountain, Varāmin.

Extralimital distribution: Ukraine, Transcaucasia, Turkey, Central Asia.

***Hyalesthes obsoletus* Signoret, 1865**

*Hyalesthes obsoletus* Signoret, 1865: 128; (Dlabola (1971a, 1972 [listed], 1981, 1994), Behdad (1988), Kheyri (1989), Mirzayans (1995), Abaii (2000), Lashkari et al. (2009).

Iran localities: Āb Āsk, Andimeshk, Behshahr, Evin, Firuzkuh, Gorgān, Hafttappeh, Karaj, Marand, Paskuh, Rezvāndeh, Sanandaj, Sufiān, Tākestān- Karaj Rd, Tochāl Mountain, Yāsuj, Ziārān.

Extralimital distribution: North Africa, Southern, Western and Eastern Europe, Northern parts of Western Asia, Afghanistan and Central Asia.

***Hyalesthes philesakis*Hoch 1986**

*Hyalesthes philesakis* Hoch 1986: 95; Dlabola (1994)

Iran localities: Masiri.

Extralimital distribution : Moldova, Ukraine, Turkey.

***Hyalesthes restultus* Dlabola, 1994**

*Hyalesthes restultus* Dlabola, 1994: 44

Iran localities: Komehr.

***Hyalesthes scotti* Ferrari, 1882**

*Hyalesthes scotti* Ferrari, 1882: 82; Dlabola (1994).

*Hyalesthes luteipes* Fieber. var. *scotti* Ferrari; (Linnavuori, 1962a).

Iran localities: Dasht, Gazanak, Sufiān, Tochāl Mountain.

Extralimital distribution: Western and central Mediterranean region, perhaps also in southern parts of central Europe. **(**Holzinger et al. 2003).

***Hyalesthes zabolicus* Dlabola, 1985**

*Hyalesthes zabolicus* Dlabola, 1985: 121; Hoch and Remane 1985 [listed].

Iran localities: Zāboli.

***Oliarellus fulvus* (Kusnezov, 1935)**

*Hyalesthes fulvus* Kusnezov, 1935: 213

*Oliarus fulvus* (Kusnezov 1935); Nast (1972) [listed], Lashkari et al*.* (2009).

*Pseudoliarus circularis* Dlabola, 1981: Dlabola (1981).

*Oliarellus fulvus*; Dlabola (1985) [listed].

Iran localities: Estahbān, Ghazvin, Golestān province, Robāt-e Tork.

Extralimital distribution: Central Asia.

***Oliarus convergens* Melichar, 1902**

*Oliarus convergens* Melichar, 1902a: 87; Dlabola (1981) [listed].

Iran localities: Kermān province.

Comment: the status of this species is unclear.

***Oliarus nigrofurcatus* Signoret, 1884**

*Oliarus nigrofurcatus* Signoret 1884: 71; Melichar (1902a), Farahbakhsh (1961) [listed], Nast (1972) [listed], Dlabola (1972) [listed].

Iran localities: Nehbandān.

Extralimital distribution: Transcaucasus, Iraq, Afghanistan and Central Asia.

Comments: The identity of this species is unclear.

***Pentastira bahtiaricus* (Dlabola, 1981)**

*Oliarus bahtiaricus* Dlabola, 1981: 144;

*Pentastira bahtiaricus*; comb. n. in Dlabola (1988).

Iran localities: Marg-e-Malek

***Pentastira major* Kirschbaum, 186*8***

*Pentastira major* Kirschbaum, 1868: 44

*Oliarus major* (Kirschbaum, 1868); Dlabola (1981), Mirzayans (1995).

*Pentastira major*; (Dlabola, 1988).

Iran localities: Akhlamad, Āmol, Behshahr, Dasht-e Moghān, Divāndareh, Gazanak, Ghāemshahr, Gharechaman, Gorgān, Kahurak, Karaj, Nowshahr, Rāmiān, Sanandaj, Shemirān, Silvāneh, Zāhedān, Zoshk.

Extralimital distribution: Southern and Eastern Europe, North parts of Western Asia.

***Pentastira superspicata* Dlabola, 1985**

*Pentastira superspicata* Dlabola, 1985: 119

Iran localities: Kāzerun, Masiri.

***Pentastira shul* (Dlabola, 1985)**

*Reptalus shul* Dlabola, 1985: 115; Dlabola (1985).

*Pentastira shul*; comb. n. in Dlabola (1988).

Iran localities: Kāzerun, Masiri, Miāneh, Shul, Yāsuj.

***Pentastiridius (Pentastiridius) leporinus* (Linnaeus, 1761)**

*Cicada leporina* Linnaeus, 1761: 242

*Oliarus leporinus*; (Dlabola (1971b, 1981).

*Flata pallens* Germar, 1821; synonymized by Nast (1986).

*Oliarus pallens* (Germar, 1821); Melichar (1902a), (Dlabola (1960a, 1971a, 1972 [listed], 1981), Farahbakhsh (1961) [listed], Nast (1972) [listed].

*Reptalus pallens* (Germar, 1821); Mirzayan (1995).

*Pentastiridius leporinus*; Nast (1986), Mirzayans (1995).

Iran localities: Ābādeh, Albāji, Bampur, Bazmān, Birjand, Chābahār, Dālaki, Evin, Gambuyeh, Gāvbandi, Gharechaman, Hafttappeh, Hamidieh, Hāresābād, Hashtpar, Irānshahr, Kandovān (Māzandarān), Marand, Miāneh- Zanjān Rd, Minushahr, Mollāsāni, Shādegān, Shieh, Susangerd, Suza, Tabriz, Varāmin, Zābol.

Extralimital distribution: North Africa, Europe, Northern parts of Western Asia, Afghanistan, Central and Eastern Asia.

***Pseudoliarus fuscofasciatus* (Melichar, 1902)**

*Oliarus fuscofasciatus* Melichar, 1902a: 88; Nast (1972) [listed], Dlabola (1972 [listed], 1981), Mirzayans (1995).

*Pseudoliarus fuscofasciatus*; Dlabola 1981.

Iran localities: Ābādeh, Bazmān, Bidruyeh, Dālaki, Geno, Ghom, Isin, Jahrom, Jiroft, Mollāsāni, Zābol.

Extralimital distribution: North east Africa, Yemen, Iraq, Transcaucasia.

***Pseudoliarus palestinensis* (Linnavuori, 1962)**

*Oliarus fuscofasciatus palestinensis* Linnavuori, 1962a: 3

*Pseudoliarus palestinensis*; Dlabola (1985).

Iran localities: Ahvāz, Jāsk, Kahkom.

Extralimital distribution: Saudi Arabia (Dlabola 1980c), Turkey (Demir 2008).

***Reptalus (Trepalus) cuspidatus* (Fieber, 1876)**

*Oliarus cuspidatus* Fieber, 1876: 215; Dlabola (1981).

*Reptalus (Trepalus) cuspidatus*; new subgenus in Emeljanov (1995).

Iran localities: Jahrom.

Extralimital distribution: Southern, Western and Eastern Europe, Turkey, Central Asia.

***Reptalus (Trepalus) rufocarinatus* (Kusnezov, 1937)**

*Oliarus quinquecostatus var. rufocarinatus* Kusnetzov, 1937: 168

*Oliarus bitinctus* Dlabola, 1961a: 254; Dlabola (1981).

*Reptalus bitinctus*; Dlabola (1985), Kheyri (1989), Mirzayans (1995).

*Oliarus concolor* Fieber, 1876; Mirzayans et al. (1976).

Iran localities: Dasht, Esfahān, Gholhak, Ghom- Esfahān Rd, Ghom, Karaj.

Extralimital distribution: North Africa, Southern, Western and Eastern Europe, Turkey, Afghanistan, Central and Eastern Asia.

Comments: *Oliarus concolor* Fieber, 1876 is taxonomically unclear, and apparently often interpreted by various author in various manners without having studied the (type material). Dlabola (1988): 64 lists it as a separate species. Also *Oliarus bitinctus* Dlabola has had a mixed history, synonymized with *rufocarinatus* by Emeljanov (1978), revived as separate species in *Setapius* by Dlabola (1988) and now as a synonym of *rufocarinatus* in *Reptalus (Trepalus)* by Emeljanov (1995).

***Reptalus eremicus* Dlabola, 1985**

*Reptalus eremicus* Dlabola, 1985: 114; Mirzayans (1995).

Iran localities: Marand, Mashhad, Masiri, Nagsh-e Rostam, Paskuh, Rafsanjān, Robāt-e Tork, Shirāz, Yāsuj.

***Reptalus melanochaetus* (Fieber, 1876)**

*Oliarus melanochaetus* Fieber, 1876: 198; Dlabola (1981), Behdad (1988), Mirzayans (1995), Abaii (2000).

*Reptalus melanochaetus*; comb. n. in Emeljanov (1972a).

Iran localities: Bābol- Ghāemshahr, Karaj, Leshtar, Tonekābon.

Extralimital distribution: Southern, Western, and Eastern Europe, Turkey, Central Asia.

***Reptalus quinquecostatus* (Dufour, 1833)**

*Cixius quinquecostatus* Dufour 1833: 224

*Reptalus quinquecostatus*; comb. n. in Emeljanov (1972b).

*Oliarus quinquecostatus* (Dufour 1833); Behdad (1988), Abaii (2000).

Iran localities: Unknown locality (Northern and central provinces).

Extralimital distribution: Southern, Western and Eastern Europe, Turkey, Central and Eastern Asia.

Comment: The identity of this record should be checked since it is part of a complex of several closely related species.

***Reptalus reductus* Dlabola, 1994**

*Reptalus reductus* Dlabola, 1994: 53; Dlabola (1994).

Iran localities: Bāft.

***Reptalus ziaran* Dlabola, 1985**

*Reptalus ziaran* Dlabola, 1985: 118

Iran localities: Miāneh, Ziārān.

***Setapius barajus* (Dlabola, 1957)**

*Oliarus barajus* Dlabola, 1957b: 24; Dlabola (1981), Mirzayans (1995)

*Setapius barajus*; comb. n. in Dlabola (1988).

Iran localities: Irānshahr, Varāmin.

Extralimital distribution: Transcaucasia, Turkey, Afghanistan.

***Setapius lindbergi* (Dlabola, 1957)**

*Oliarus lindbergi* Dlabola, 1957b: 23; Linnavuori (1962a) [listed], Nast (1972) [listed], Dlabola (1972 [listed], 1981).

*Setapius lindbergi*; comb. n. in Dlabola (1988), Kheyri (1989), Mirzayans (1995).

*Reptalus lindbergi* (Dlabola, 1960); Kheyri (1989), Mirzayans (1995).

Iran localities: Āb Āsk, Bampur, Fasā, Gorgān, Heirān, Jiroft, Marand, Marg-e-Malek, Sarāvān, Shirāz, Tochāl Mountain, Zābol.

Extralimital distribution: Afghanistan, Cyprus, Iraq, Israel, Turkey, Dagestan, Uzbekistan (Nast 1972) Egypt (Dlabola 1974b), Saudi Arabia (Dlabola 1980c).

**Family Delphacidae**

**Subfamilies and tribes follow Asche (1985)**

**Subfamily Asiracinae**

***Asiraca clavicornis* (Fabricius, 1794)**

*Cicada clavicornis* Fabricius, 1794: 41; Karimzadeh Esfahani et al. (1998), Behdad (1988), Mirzayans (1995) Abaii (2000).

*Asiraca flavicornis* (sic); (Dlabola (1981, 1984).

Iran localities: Esfahān province, Goleestān province, Karaj, Markazi province.

Extralimital distribution: North Africa, Europe, Northern parts of Western Asia, Afghanistan, Central and Eastern Asia.

**Subfamily Kelisinae**

***Kelisia praecox* Haupt, 1935**

*Kelisia praecox* Haupt, 1935: 133; Dlabola (1981), Lashkari et al. (2009)

Iran localities: Tochāl Mountain, Golestān province.

Extralimital distribution: Germany, Eastern Europe, Central and Eastern Asia.

***Kelisia ribauti* Wagner, 1938**

*Kelisia ribauti* Wagner, 1938: 12; Dlabola (1981), Mirzayans (1995).

Iran localities: Evin.

Extralimital distribution: Western, Northern and Eastern Europe, North parts of Westsern Asia, Afghanistan, Central Asia, Eastern Asia and Russian Far East.

**Subfamily Stenocraninae**

***Stenocranus major* (Kirschbaum, 1868)**

*Delphax major* Kirschbaum, 1868: 211

*Stenocranus major*; Dlabola (1981).

Iran localities: Golestān province.

Extralimital distribution: Europe, Afghanistan.

**Subfamily Delphacinae**

**Tribe Tropidocephalini**

***Tropidocephala prasina* Melichar, 1902**

*Tropidocephala prasina* Melichar, 1902a: 90

*Tropidocephala prasina lateralis* Melichar, 1902a: 90; Nast (1972) [listed], Dlabola (1981) [listed].

Iran localities: Bazmān.

**Tribe Delphacini**

***Bostaera bolivari* (Melichar, 1901)**

*Delph acodes bolivari* Melichar, 1901: 56

*Pseudaraeopus bolivari* (Melichar, 1901); Nast (1972) [listed].

*Bostaera bolivari*; (Dlabola (1960a, 1981 [listed])., Mirzayans (1995).

Iran localities: Chābahār, Geno, Irānshahr, Isin, Ziārat.

Extralimital distribution: North West Africa, Southern Europe, Israel.

***Calligypona reyi* (Fieber, 1866)**

*Delphax reyi* Fieber, 1866: 527

*Calligypona reyi*; Dlabola (1981).

Iran localities: Bandar Anzali.

Extralimital distribution: Europe, Turkey, Central Asia.

***Changeondelphax velichkovskyi* (Melichar, 1913)**

*Euidella velitchkovskyi* Melichar, 1913: 6 (type species of *Changeondelphax* Kwon, 1982*)*

*Calligypona oriens* Dlabola, 1961a: 276 (synonymized by Anufriev 1977).

*Chloriona oriens*; Dlabola (1981).

*Changeondelphax velichkovskyi*; comb. n. in Kwon (1982).

Iran localities: Ghāemshahr.

Extralimital distribution: South Eastern Europe, North Caucasus Eastern Asia.

***Chloriona clavata* Dlabola, 1960**

*Chloriona clavata* Dlabola, 1960b: 1; Dlabola (1981).

Iran localities: Gorgān, Evin.

Extralimital distribution: Kyrgyzia (Novikov et al. 2006).

***Chloriona unicolor* (Herrich-Schäffer, 1835)**

*Delphax unicolor* Herrich-Schäffer, 1835: 66

*Chloriona unicolor*; Melichar (1902a), Dlabola (1968 [listed], 1981), Mirzayans (1972), Nast (1972) [listed].

Iran localities: Aliābād (Ghom), Evin, Gambuyeh, Marand, Temin

Extralimital distribution: North Africa, Europe, Turkey, Central Asia

***Conomelus* sp*.***

*Conomelus* sp*.*; Dlabola (1981).

Iran localities: Tochāl Mountain.

***Delphacodes audrasi* Ribaut, 1954**

*Delphacodes audrasi* Ribaut, 1954: 180

*Delphacodes linnavuorii* Le Quesne, 1960: 160; Dlabola (1981), Mirzayans (1995).

Iran localities: Bidruyeh, Gambuyeh, Minushahr, Sufiān.

Extralimital distribution: Southern parts of Eastern Europe, Southern Europe, Turkey, Israel.

Comments: Asche and Remane (1983) discuss the status of *Delphacodes mulsanti* Fieber and suggested that both *Delphacodes audrasi* and *Delphacodes linnavuorii* are likely to be synonyms of *mulsanti*.

***Delphacodes ornatipennis* (Haupt, 1927)**

*Megamelus ornatipennis* Haupt, 1927: 9; Dlabola (1964) [listed], 1965c [listed], 1960a, 1972).

*Delphacodes ornatipennis*; Asche and Remane (1983).

Iran localities: Irānshahr, Shādegān.

Extralimital distribution: East Mediterranean, Afghanistan.

Comments: Likely to become a synonym of *Delphacodes mulsanti* after further investigation (M. Asche pers comm.).

***Delphax inermis* Ribaut, 1934**

*Delphax inermis* Ribaut, 1934: 281; Dlabola (1977b [listed], 1994), Mirzayans (1995).

Iran localities: Borāzjān, Gambuyeh, Konārdān, Minushahr.

Extralimital distribution: North West Africa, Southern and Western Europe, East Mediterranean, Afghanistan.

***Dicranotropis hamata* (Boheman, 1847)**

*Delphax hamata* Boheman, 1847: 45

*Dicranotropis hamata*; Dlabola (1981).

Iran localities: Lajrān.

Extralimital distribution: North Africa, Europe, North Asia, Central Asia.

***Euides caspiana* (Dlabola, 1961)**

*Euidella caspiana* Dlabola, 1961a: 268

*Euides caspiana*; Dlabola (1981).

Iran localities: Bandar Anzali.

Extralimital distribution: Dagestan (Nast, 1972), Turkey (Demir, 2008).

***Falcotoya minuscula* (Horváth, 1897)**

*Delphax minuscula* Horváth, 1897b: 622

*Calligypona minuscula* (Horváth, 1897); Dlabola (1960a).

*Toya minuscula* (Horváth, 1897): Dlabola (1981), Dlabola (1972) [listed].

*Falcotoya minuscula*; Barkhordari et al. (1981) [listed].

Iran localities: Bandar Anzali, Irānshahr, Robāt-e Tork.

Extralimital distribution: North East Africa, France, Eastern Europe, Turkey, Afghanistan and Central Asia.

***Gravesteiniella boldi* (Scott, 1870)**

*Liburnia boldi* Scott, 1870: 68

*Gravesteiniella boldi*; Dlabola (1981).

Iran localities: Golestān province.

Extralimital distribution: Western, Northern and Estern Europe, Cyprus, Central Northern and Eastern Asia.

***Halmyra aeluropodis* (Emeljanov, 1964)**

*Calligypona aeluropodis* Emeljanov, 1964: 9

*Halmyra aeluropodis*; (Dlabola (1974a, 1981).

Iran localities: Robāt-e Tork.

Extralimital distribution: Kazakhstan (Nast, 1972), Greece (Drosopoulos et al.1983).

***Herbalima eforiae* (Dlabola, 1961)**

*Calligypona eforiae* Dlabola, 1961b: 314

*Unkanodes eforiae*; comb. n. in Dlabola (1974a).

*Herbalima eforiae*; comb. n. in Emeljanov (1977).

Iran localities: Eynvarzān.

Extralimital distribution: Romania, Kazakhstan (Nast ,1972) Kyrgyzia (Novikov et al. 2006).

***Javesella pellucida* (Fabricius, 1794)**

*Fulgora pellucida* Fabricius, 1794: 7

*Calligypona marginata* (Fabricius, 1794); Dlabola (1960a).

*Javesella pellucida*; Dlabola (1981), Mirzayans (1995).

Iran localities: Anbarābād, Chābahār, Evin, EynvarzānIrānshahr, Sufiān.

Extralimital distribution: North of Africa, Europe, Turkey, Afghanistan, Central, Eastern and Northern Asia.

***Laodelphax striatellus* (Fallén, 1826)**

*Delphax striatellus* Fallén, 1826: 75

*Laodelphax striatellus* (Fallén, 1826); (Dlabola (1971a, 1981), Nast (1972) [listed], Ahmadi et al. (1986), Kheyri (1989), Mirzayans (1995), Karimzadeh Esfahani et al. (1998), Haghshenas and Khajehali (2000), Nematollahi and Khajehali (2000), Khajehali et al. (2001), Yarmand et al. (2006), Lashkari et al. (2009).

*Laodelphax striatella*; Mirzayans et al. (1976).

Iran localities: Ahvāz, Aliābād (Jahrom), Āmol, Asālem, Bājgāh, Bandar Anzali, Behshahr, Behshahr- Sāri, Bidruyeh, Bidzard, Chenārshāhijān, Dārāb, Darband, Dasht, Eskandari, Estahbān, Evin, Eynvarzān, Fasā, Galugāh- Eslāmābād, Gambuyeh, Gazanak, Gholhak, Gorgān, Hashtpar, Jahānābād Sepidān, Jiroft, Karaj, Marand, Miāneh- Gharechaman, Minushahr, Nurābād, Parehsar, Robāt-e gharebil- Dowlatābād, Sanandaj, Sāri- Bābolsar , Sarvestān, Shahdād, Shahr-e Kord, Shirāz, Sufiān, Tabriz, Veresk, Zarghān.

Extralimital distribution: North Africa, Europe, Western Asia, Afghanistan, Central, Northern and Eastern Asia.

***Matutinus putoni* (Costa, 1888)**

*Kelisia putoni* Costa, 1888: 16

*Calligypona typhae* Lindberg, 1960: 15 (syn. Asche and Remane, 1982); Dlabola (1981)

*Matutinus putoni*;Mirzayans (1995).

Iran localities: Anbarieh, Minushahr, Shush.

Extralimital distribution: Italy, North Africa, Israel.

***Muirodelphax amol* Dlabola, 1981**

*Muirodelphax amol* Dlabola, 1981: 154

Iran localities: Āmol.

***Muirodelphax aubei* (Perris, 1857)**

*Delphax aubei* Perris, 1857: 170

*Muirodelphax aubei*; Dlabola (1981), Lashkari et al. (2009)

Iran localities: Damāvand, Golestān province, Lajrān, Marand, Tochāl Mountain.

Extralimital distribution: North Africa, Europe, Turkey, Central and Eastern Asia.

***Pseudaraeopus curtulus* Dlabola, 1960**

*Pseudaraeopus curtulus* Dlabola, 1960a: 3; Dlabola (1981) [listed], Nast (1972) [listed].

Iran localities: Irānshahr.

***Pseudaraeopus iranicus* Dlabola, 1960**

*Pseudaraeopus iranicus* Dlabola, 1960a: 4; Dlabola (1981) [listed], Nast (1972) [listed].

Iran localities: Irānshahr.

***Ribautodelphax hyrcanus* Dlabola, 1981**

*Ribautodelphax hyrcanus* Dlabola, 1981: 157; Mirzayans (1995)

Iran localities: Golestān province, Hashtpar.

***Sardia rostrata* Melichar, 1903**

*Sardia rostrata* Melichar, 1903: 96; Dlabola (1981), Mirzayans (1995)

Iran localities: Minushahr.

Extralimital distribution: widespread in Asia and Australasia.

***Sogatella furcifera* (Horváth, 1899)**

*Delphax furcifera*Horváth 1899: 372

*Sogatella furcifera*; Lashkari et al. (2009).

Iran localities: Golestān province.

Comments: Asche and Wilson (1990) recorded no specimens of this species in Europe or Africa. It is very likely that this record refers to another species.

Extralimital distribution: Eastern Palaearctic (Asche and Wilson, 1990).

***Sogatella vibix* (Haupt, 1927)**

*Liburnia vibix* Haupt, 1927:13

*Calligypona vibix* (Haupt, 1927); Dlabola (1960a).

*Sogatella suezensis* Matsumura 1910; Linnavuori (1964) misdetermination (not *suezensis* Matsumura, 1910); Ahmadi et al. (1986), Kheyri (1989), Mirzayans (1995), Nast (1972) [listed].

*Sogatella vibix*; (Dlabola (1965c, 1971a, 1972), Karimzadeh Esfahani et al. (1998), Lashkari et al. (2009).

Iran localities: Albāji, Anbarābād, Bājgāh, Bidruyeh, Dārāb, Esfahān province, Estahbān, Fasā, Firuzābād, Golestān province, Irānshahr, Isin, Jiroft, Minushahr, Nurābād, Sarvestān, Shabestar.

Extralimital distribution: North of Africa, North parts of Western Asia, Afghanistan.

Comments: Asche and Wilson (1990) provide full synonymy and localities.

***Toya propinqua* (Fieber, 1866)**

*Delphax propinqua* Fieber, 1866: 525

*Calligypona propinqua* (Fieber, 1866); Dlabola (1960a).

*Toya propinqua*; Ahmadi et al. (1986), Dlabola (1981), Kheyri (1989), Lashkari et al. (2009); Mirzayans (1995), Nast (1972) [listed].

Iran localities: Āb Āsk, Anbarābād, Andimeshk, Bājgāh, Bandar Anzali, Eskandari, Evin, Eynvarzān, Fasā, Gholhak, Gorgān, Hashtpar, Irānshahr, Isin, Jiroft, Kāzerun, Marand, Minushahr, Rudbārak, Sanandaj, Sepidān, Shirāz, Tochāl Mountain, Zarghān Extralimital distribution: North Africa, Southern, Western and Eastern Europe, Western Asia, Afghanistan, Central Asia and Japan.

***Unkanodes latespinosa* (Dlabola, 1957)**

*Calligypona latespinosa* Dlabola, 1957a: 275

*Unkanodes latespinosa*; (Dlabola (1971a, 1981), Mirzayans (1995).

Iran localities: Damāvand, Eskandari, Evin, Gholhak, Marand, Miāneh – Gharechaman, Orumieh, Robāt-e gharebil, Sanandaj, Shahdād.

Extralimital distribution: Turkey, Afghanistan and Mongolia.

***Unkanodes tanasijevici* (Dlabola, 1965)**

*Elymodelphax tanasijevici* Dlabola, 1965a: 658

*Unkanodes tanasijevici*; Ahmadi et al. (1986).

Iran localities: Akbar ābād, Bājgāh, Zarghān.

Extralimital distribution: Turkey-Anatolia, Greece, Romania, (Asche,1982), Yugoslavia.

**Family Derbidae**

**Tribe Cenchreini**

***Malenia isinica* Dlabola, 1986**

*Malenia isinica* Dlabola, 1986: 173

Iran localities: Isin.

***Malenia masirica* Dlabola, 1986**

*Malenia masirica* Dlabola, 1986: 173

Iran localities: Dehbakri, Kermānshāh (Yazd province), Kushk, Masiri, Sheikh-e Mahalleh, Yāsuj.

***Malenia sarmatica* Anufriev, 1966**

*Malenia sarmatica* Anufriev, 1966: 46; Dlabola (1981), Mirzayans (1995).

Iran localities: Hashtpar, Lāhijān, Tonekābon, Zirāb.

Extralimital distribution: Ukraine, Transcaucasia.

**Tribe Zoraidini**

***Proutista jezeki* Dlabola, 1981**

*Proutista jezeki* Dlabola, 1981: 161

Iran localities: Rāsk.

**Family Dictyopharidae**

**Subfamily Dictyopharinae**

**Subgenera of *Dictyophara* follow Emeljanov (2004)**

***Dictyophara (Ancylocrius) albata* Dlabola & Heller, 1962**

*Dictyophara albata* Dlabola & Heller, 1962: 2; Nast (1972) [listed], Dlabola (1981), Behdad (1988), Mirzayans (1995), Abaii (2000), Mozaffarian and Emeljanov (2010) [listed].

Iran localities: Chāhkuh, Estahbān, Geno, Hichān, Irānshahr, Jahrom, Jiroft, Kahurestān, Kāzerun, Nikshahr, Sendarak.

***Dictyophara (Ancylocrius) exoptata* Dlabola & Heller, 1962**

*Dictyophara exoptata* Dlabola & Heller, 1962: 1; Nast (1972) [listed], Mirzayans et al. (1976) Dlabola (1981), Mirzayans (1995), Mozaffarian and Emeljanov (2010) [listed].

Iran localities: Aliābād (Jahrom), Chāhkuh, Dasht-e arjan, Geno, Irānshahr, Isin, Jiroft, Kahurestān, Kāzerun, Konārdān, NikshharSendarak, Shirāz.

***Dictyophara (Chanithus) avocetta* Oshanin, 1879**

*Dictyophara avocetta* Oshanin, 1879: 131; Melichar, 1902a, Nast (1972) [listed], Dlabola (1981) [listed], Mozaffarian and Emeljanov (2010) [listed].

Iran localities: Anārak.

Extralimital distribution: Azerbaijan, Eastern parts of Central Asia.

***Dictyophara (Chanithus) hastata* Kusnezov, 1929**

*Dictyophara hastate* Kusnezov, 1929; Mozaffarian and Emeljanov (2010) [listed].

***Dictyophara (Chanithus) kazeruna* Dlabola, 1986**

*Dictyophara kazeruna* Dlabola, 1986: 179, Mozaffarian and Emeljanov (2010) [listed].

Iran localities: Kāzerun.

***Dictyophara (Conopenchus) pazukii* (Dlabola, 1984)**

*Philotheria pazukii* Dlabola, 1984: 25

Iran localities: Kamandān.

***Dictyophara (Dictyophara) asiatica* Melichar, 1912**

*Dictyophara asiatica* Melichar, 1912: 118; Dlabola (1981), Behdad (1988), Abaii (2000), Mozaffarian and Emeljanov (2010) [listed].

Iran localities: Eynvarzān, Gazanak.

Extralimital distribution: Northern parts of Western Asia.

***Dictyophara (Dictyophara) europaea* (Linnaeus, 1767)**

*Fulgora europaea* Linnaeus, 1767: 704

*Dictyophara europaea*; Mirzayans et al. (1976), Dlabola (1981), Behdad (1988), Mirzayans (1995), Haghshenas and Khajehali (2000), Abaii (2000), Khajehali et al. (2001), Mozaffarian and Emeljanov (2010) [listed].

Iran localities: Ahvāz, Bābol- Ghāemshahr, Bandar Anzali, Behshahr, Chahār mahāl- Bakhtiāri province, Eskandari, Fasā, Feizābād, Hafttappeh, Jahrom, Kāmyārānv, Kandovān (Māzandarān), Khorramābād, Marand, Mollāsāni, Tabriz, Tonekābons, Varāmin, Zirāb.

Extralimital distribution: North Africa, Western, Eastern and Southern Europe, northern parts of Western Asia, Afghanistan, southern parts of Central Asia and Eastern Asia.

***Dictyophara (Euthremma) hoberlandti* Dlabola, 1974**

*Dictyophara hoberlandti* Dlabola, 1974a: 36; Dlabola (1981), Mirzayans et al*.* (1976), Behdad (1988), Mirzayans (1995), Abaii (2000), Mozaffarian and Emeljanov (2010) [listed].

*Dictyophara (Euthremma) hoberlandti*; Emeljanov (2004).

Iran localities: Eynvarzān, Gāvkoshak, Kāzerun, Orumieh, Sepidān, Sisakht.

***Raivuna iranica* (Linnavuori, 1962)**

*Dictyophara iranica* Linnavuori, 1962a: 7; Nast (1972) [listed], Dlabola (1981), Mirzayans (1995), Mozaffarian and Emeljanov (2010) [listed].

Iran localities: Anbarābād, Chāhkuh, Dālaki, Evin, Ghom Lake, Hafttappeh, Hasanlangi, Irānshahr, Isin, Jiroft, Kandovān (Māzandarān), Marand, Mollāsāni, Nikshahr, Pishin, Rāsk, Sendarak, Sisakht, Yazd, Zābol.

Extralimital distribution: Turkey (Demir, 2008).

***Raivuna pallida* (Donovan, 1800)**

*Fulgora pallida* Donovan 1800: 1

*Chanithus pallidus* (= *striata* Oshanin, 1879); Dlabola (1960a).

*Raivuna pallida*; Mozaffarian and Emeljanov (2010) [listed].

Iran localities: Anbarābād, Bampur, Irānshahr

Extralimital distribution: North Africa, Southern parts of Western Asia, Transcaucasia, Central and Eastern Asia.

Comments: Linnavuori (1962a) regarded *Chanithus* as a subgenus of *Dictyophara*. Due to problems with the identity of this species literature records may refer to a number of species.

***Raivuna striata* (Oshanin, 1879)**

*Raivuna striata* (Oshanin, 1879); Mozaffarian and Emeljanov (2010) [listed].

Iran localities: Unknown locality.

**Subfamily Orgeriinae**

***Kumlika mandrita* Emeljanov, 1997**

*Kumlika mandrita* Emeljanov, 1997: 89

Iran localities: Sabzevār.

***Nymphorgerius convergens* Emeljanov, 1972**

*Nymphorgerius convergens* Emeljanov, 1972a: 25; Dlabola (1979b).

Iran localities: Shāhkuh-e Pāiin.

***Nymphorgerius emeljanovi* Dlabola, 1979**

*Nymphorgerius emeljanovi* Dlabola, 1979b: 242

Iran localities: Tochāl Mountain, Ziārān.

***Nymphorgerius mullah* Dlabola, 1979**

*Nymphorgerius mullah* Dlabola, 1979b: 241

Iran localities: Ziārān.

***Nymphorgerius plotnikovi* Kusnezov, 1929**

*Nymphorgerius plotnikovi* Kusnezov, 1929: 326; Emeljanov (1997).

Iran localities: Bojnurd.

Extralimital distribution: Kazakhstan, Turkmenia (Nast, 1972).

***Nymphorgerius rostratus* Emeljanov, 2009**

*Nymphorgerius rostratus* Emeljanov, 2009: 283

Iran localities: Sangrud.

***Tigrahauda ototettigoides* (Oshanin, 1913)**

*Orgerius ototettigoides* Oshanin, 1913: 140

*Tigrahauda ototettigoides*; Emeljanov (1997).

Iran localities: Esfarāyen, Kāhe.

Extralimital distribution: Central Asia.

**Family Flatidae**

***Bahuflata punctata* Dlabola, 1979**

*Bahuflata punctata* Dlabola, 1979a: 230

Iran localities: Bāhukalāt, Sirik.

***Derisa atratula* Melichar, 1902**

*Derisa atratula* Melichar, 1902a: 103; (Dlabola (1960a, 1981), Nast (1972) [listed], Mirzayans (1995), Medler (2003).

Iran localities: Sirjān, Isin, Anbarābād, Bampur, Bazmān, Nikshahr, Bāhukalāt, Chābahār, Tis.

***Eurima astuta* Melichar, 1902**

*Eurima astuta* Melichar, 1902a: 102; Dlabola (1981) [listed], Nast (1972) [listed], Mirzayans (1995).

Iran localities: Bazmān, Kahurestān, Sendarak.

Extralimital distribution: Israel (Nast 1972) Saudi Arabia (Dlabola 1979d).

***Mesophantia kanganica* Dlabola, 1983**

*Mesophantia kanganica* Dlabola, 1983: 469 in Krampl and Dlabola (1983).

Iran localities: Bānuchārehar, Bojnurd, Hessār, Jiroft, Kangān, Nehbandān, Nosratābad, Sabzevār.

***Mesophantia pallens* Melichar, 1902**

*Mesophantia pallens* Melichar, 1902b: 18; Abaii (2000), Barkhordari et al. (1981), (Dlabola (1981, 1983a), Medler (2003), Mirzayans (1995), Mirzayans et al. (1976), Nast (1972) [listed], Rajabi (1989).

Iran localities: Ābādeh, Bojnurd, Darpahn, Estahbān, Evin, Gāvkoshak, Izadkhāst, Jahrom, Kāshān, Kāzerun, Khosro- Shirin, Mashhad-e Ardehāl, Mohammadābād, Nishābur, Rafsanjān, Sangān, Shāhrud, Sisakht, Yāsuj.

***Mesophantia sabzevaranica* Dlabola, 1983**

*Mesophantia sabzevaranica* Dlabola, 1983: 466 in Krampl and Dlabola, (1983); Mirzayans (1995).

Iran localities: Denā mt (west slope), Doborji, Estahbān, Ferdows-e Esfandaghe, Gāvkoshi, Geno, Ghaderābād, Jahrom, Jiroft, Kāzerun, Kermān, Kuhenjān, Māhārlu, Miānjangal, Mohammadābād, Nishābur, Posht- e kuh, Sarāvān, Sarbāz, Shul, Sisakht.

***Mesophantia tisina* Dlabola, 1983**

*Mesophantia tisina* Dlabola, 1983: 468 in Krampl and Dlabola (1983).

Iran localities: Dehbakri, Tis.

***Persepolia columbaria* Dlabola & Safavi, 1972**

*Persepolia columbaria* Dlabola & Safavi, 1972: 2; Dlabola and Safavi (1972), (Dlabola (1974a, 1981), Mirzayans et al*.* (1976), Behdad (1988), Rajabi (1989), Mirzayans (1995), Abaii (2000).

Iran localities: Kāzerun, Nurābād, Shirāz, Yāsuj.

Extralimital distribution: Saudi Arabia (Dlabola, 1980c).

***Persepolia jasmuriana* Dlabola, 1982**

*Persepolia jasmuriana* Dlabola, 1982b: 165

Iran localities: Bāhukalāt, Bārgāh, Bazmān, Geno, Isin, Rudān, Sarāvān, Tang-e Sarheh.

***Persepolia secunda* Dlabola, 1981**

*Persepolia secunda* Dlabola, 1981: 190

Iran localities: Zāboli.

***Persepolia servadeina* Dlabola, 1982**

*Persepolia servadeina* Dlabola, 1982b: 163

Iran localities: Bazmān, Zāboli, Pākuh, Sarāvān, Tang-e Sarheh, Zāhedān, Kermānshāh (Yazd province), Khutanābād, Kermān, Māhān, Golbāf.

***Phantia borazianica* Dlabola, 1989**

*Phantia borazianica* Dlabola, 1989: 44

Iran localities: Borāzjān.

***Phantia christophii* Rusiecka, 1902**

*Phantia christophii* Rusiecka, 1902: 423; Melichar (1902a), Dlabola (1972), Nast (1972) [listed].

Iran localities: Temin.

Extralimital distribution: Afghanistan, Turkmenia.

***Phantia crucispina* Dlabola, 1989**

*Phantia crucispina* Dlabola, 1989: 42

Iran localities: Zāhedān.

***Phantia cylindricornis* Melichar, 1902**

*Phantia cylindricornis* Melichar, 1902a: 99; (Dlabola (1960a, 1972 [listed], 1989), Nast (1972) [listed], Medler (2003).

Iran localities: Anbarābād, Bāhukalāt, Chābahār, Espakeh, Hasanlangi, Irānshahr, Mināb, Ziārat.

Extralimital distribution: Afghanistan and Turkmenia.

***Phantia denasuta* Dlabola, 1989**

*Phantia denasuta* Dlabola, 1989: 40

Iran localities: Bāhukalāt, Bampur, Bandar Chārak, Hasanlangi, Jāsk.

***Phantia ferganensis* Dubovsky, 1966**

*Phantia ferganensis* Dubovsky, 1966: 75; Dlabola (1989).

Iran localities: Bāhukalāt, Bampur, Jāsk.

Extralimital distribution: Uzbekistan.

***Phantia finita* Dlabola, 1989**

*Phantia finita* Dlabola, 1989: 49

Iran localities: Miānjangal.

***Phantia flavida* Rusiecka, 1902**

*Phantia flavida* Rusiecka, 1902: 424; Nast (1972) [listed], Dlabola (1981) [listed].

Iran localities: Unknown locality.

Extralimital distribution: Azerbaijan.

***Phantia helleri* Linnavuori, 1962**

*Phantia helleri* Linnavuori, 1962b: 2; Nast (1972) [listed], (Dlabola (1981, 1989), Mirzayans (1995).

Iran localities: Ahram, Bāhukalāt, Bampur, Bandar Abbās, Bandar Lengeh, Bārgāh, Bazmān, Dārāb, Dārbahāre, Darzin, Darpahn, Fāriāb, Gambuyeh, Ganāveh, Gāvkoshi, Geno, Ghasr-e Ghand, Ghom, Ghotbābād, Hājiābād, Hasanlangi, Isin, Jāsk, Jiroft, Kahnuj, Kahurestān, Kamālābād, Khutanābād, Mahārlu, Mijān, Mināb, Nikshahr, Nugh, Rafsanjān, Rāsk, Rudān, Sekand, Sendarak, Shirāz, Shul, Shusf, Sirik, Tang-e Sarheh, Tis, Zeidān.

***Phantia lactea* Rusiecka, 1902**

*Phantia lactea* Rusiecka, 1902: 424; Nast (1972) [listed], Dlabola (1981) [listed].

Iran localities: Unknown locality.

***Phantia ovatospina* Dlabola, 1989**

*Phantia ovatospina* Dlabola, 1989: 43

Iran localities: Nehbandān.

***Phantia picea* Dlabola, 1989**

*Phantia picea* Dlabola, 1989: 46

Iran localities: Bāhukalāt, Bandar Abbās, Jāsk, Nikshahr.

***Phantia putoni* Rusiecka, 1902**

*Phantia putoni* Rusiecka, 1902: 423; Dlabola (1981) [listed].

Iran localities: Unknown locality.

***Phantia rubromarginata* Rusiecka, 1902**

*Phantia rubromarginata* Rusiecka, 1902: 424; Nast (1972) [listed], Dlabola (1972 [listed], 1981 [listed]).

Iran localities: Unknown locality.

Extralimital distribution: Afghanistan.

***Phantia subquadrata* (Herrich Schäffer, 1838)**

*Poeciloptera subquadrata* Herrich Schäffer, 1838: 2

*Phantia subquadrata*; Dlabola (1989).

Iran localities: Yāsuj.

Extralimital distribution: Turkey (Demir 2007).

***Phantia viridula* Puton, 1890**

*Phantia viridula* Puton, 1890: 230; Dlabola (1972) [listed], Nast (1972) [listed], Puton (1890).

Iran localities: Shāhrud.

Extralimital distribution: Armenia.

***Tisia esfandiarii* Dlabola, 1981**

*Tisia esfandiarii* Dlabola, 1981: 192

Iran localities: Tis.

***Zarudnya fusca* Melichar, 1902**

*Zarudnya fusca* Melichar, 1902a: 101; Dlabola (1960a), Nast (1972) [listed], Medler (2003).

Iran localities: Bampur, Bazmān, Irānshahr.

Extralimital distribution: Turkmenia.

***Zarudnya interstitialis* Melichar, 1902**

*Zarudnya interstitialis* Melichar, 1902a: 101; (Dlabola (1960a, 1981 [listed]), Nast (1972) [listed], Medler (2003), Mirzayans (1995)

Iran localities: Anbarābād, Bampur, Bazmān, Irānshahr, Jiroft, Kharposht, Sendarak.

Extralimital distribution: North Africa.

**Family Fulgoridae**

The genus *Dorysarthrus* was transferred from Dictyopharidae to Fulgoridae by Emeljanov (1979).

***Dorysarthrus mobilicornis* Puton, 1895**

*Dorysarthrus mobilicornis* Puton, 1895: 88; Dlabola (1981), Mirzayans (1995).

Iran localities: Bāhukalāt, Ghasr-e Ghand, Nikshahr, Sirik.

Extralimital distribution: Israel.

***Dorysarthrus simonyi* Melichar, 1912**

*Dorysarthrus simonyi* Melichar, 1912; Dlabola 1984, Mirzayans (1995).

Iran localities: Mināb.

Extralimital distribution: Saudi Arabia (Dlabola. 1979d).

***Dorysarthrus sumakovi* Oshanin, 1908**

*Dorysarthrus sumakovi* Oshanin, 1908: 471; Nast (1972) [listed].

Iran localities: Unknown locality.

Extralimital distribution: Turkmenia.

**Family Issidae Spinola, 1839**

***Anatalodus karabachicus* (Logvinenko, 1975)**

*Aeluropsis karabachica* Logvinenko, 1975: 59

*Hysteropterum ignavum* Dlabola, 1981: 179; Dlabola (1981).

*Anatolodus ignavus*; Dlabola (1982a), Mirzayans (1995).

*Anatalodus karabachicus*; Gnezdilov (2010b) [listed].

Iran localities: Ghezel-Bolāgh, Māku, Khoy, Marand, Nikshahr.

Extralimital distribution: Turkey.

***Cavatorium ardakanum* Dlabola, 1980**

*Cavatorium ardakanum* Dlabola, 1980b: 210

Iran localities: Shul.

***Cavatorium bispinatum* Dlabola, 1980**

*Cavatorium bispinatum* Dlabola, 1980b: 209

Iran localities: Sisakht, Yāsuj.

***Cavatorium quadrispinatum* Dlabola, 1980**

*Cavatorium quadrispinatum* Dlabola, 1980b: 211

Iran localities: Doborji, Fasā.

***Cavatorium sarbaz* Dlabola, 1980**

*Cavatorium sarbaz* Dlabola, 1980b: 212

Iran localities: Sekand.

***Eusarima (Nepalius) iranica* Gnezdilov & Mozaffarian, 2011**

*Eusarima (Nepalius) iranica* Gnezdilov & Mozaffarian, 2011: 457

Iran localitities: Dārābād, Tehrān.

***Inflatodus astyages* Dlabola, 1982**

*Inflatodus astyages* Dlabola, 1982a: 124

Iran localities: Ziārān.

***Inflatodus kyaxares* Dlabola, 1982**

*Inflatodus kyaxares* Dlabola, 1982a: 122

Iran localities: Ziārān.

***Inflatodus persicus (*Dlabola, 1981)**

*Hysteropterum persicum* Dlabola, 1981: 181

*Inflatodus persicus*; Mirzayans (1995).

Iran localities: Ābyek, Ghazvin, Tochāl Mountain, Zanjān.

***Inflatodus viridans* (Dlabola, 1974)**

*Hysteropterum viridatum* Dlabola, 1971a: 380 [Preoccupied]

*Hysteropterum viridans* Dlabola, 1974a: 44 (nom. nov. for *Hysteropterum viridatum* Dlabola, 1971: 380 nec Caldwell, 1945); Dlabola (1974a [listed], 1981 [listed]).

*Inflatodus viridans*; Mirzayans (1995).

Iran localities: Ghezel-Bolāgh, Varāmin.

***Iranodus amygdalinus* Dlabola, 1980**

*Iranodus amygdalinus* Dlabola, 1980b: 205; Behdad (1988), Mirzayans (1995), Abaii (2000).

Iran localities: Bānuchārehar, Chābahār, Doborji, Ferdows-e Esfandaghe, Gotbābād, Hoseinābād, Isin, Jiroft, Kāzerun, Miānjangal, Mohammadābād.

***Iranodus dumetorus (*Dlabola, 1981), comb. n.**

*Hysteropterum dumetorum* Dlabola 1979 nomen nudum. Species not listed or described

*Iranodus dumetorus (*Dlabola, 1980b); 205 nomen nudum. Species listed but not described.

*Hysteropterum dumetorum* Dlabola, 1981: 185

Iran localities: Khānehkhoreh, Shirāz, Dehbid.

Comments: The species was validly described as *Hysteropterum dumetorum* in Dlabola (1981). However, the name appeared as a new combination in Dlabola (1980b) based on the species being described in Dlabola (1979), where in fact it was not mentioned. On this basis the two earlier citations of the name must be nomina nuda. But the new combination given as *Iranodus dumetorus* was 2 years before the species was described. Accordingly we validate the name here in the combination *Iranodus dumetorus* (Dlabola, 1981).

***Iranodus khatunus* (Dlabola, 1981), comb. n.**

*Hysteropterum khatunum* Dlabola, 1979 nomen nudum. Species not listed or described

*Iranodus khatunus* Dlabola, 1979: Mirzayans (1995).

*Iranodus khatunus* (Dlabola, 1980b); 205 nomen nudum. Species listed but not described Mirzayans (1995).

*Hysteropterum khatunum* Dlabola, 1981: 187

Iran localities: Bānuchārehar, Dehpābid.

Comments: The species was validly described as *Hysteropterum khatunum* in Dlabola (1981). However, the name appeared as a new combination in Dlabola (1980b) based on the species being described in Dlabola (1979), where in fact it was not mentioned. On this basis the two earlier citations of the name must be *nomina nuda*. But the new combination given as *Iranodus khatunus* was 2 years before the species was described. Accordingly we validate the name here in the combination *Iranodus khatunus* (Dlabola, 1981).

***Iranodus nishabur* Dlabola, 1982**

*Iranodus nishabur* Dlabola, 1982a: 128

Iran localities: Birjand, Nishābur.

***Iranodus repandus* (Dlabola, 1981), comb. n.**

*Hysteropterum repandum* Dlabola, 1979 nomen nudum. Species not listed or described.

*Iranodus repandus* Dlabola, 1979; Mirzayans (1995).

*Iranodus repandum* (Dlabola, 1980b): 207 nomen nudum. Species listed but not described.

*Hysteropterum repandum* Dlabola, 1981: 183; Abaii (2000), Behdad (1988).

Iran localities: Jahrom, Kāzerun, Shirāz.

Comments: The species was validly described as *Hysteropterum repandus* in Dlabola (1981). However, the name appeared as a new combination in Dlabola (1980b) based on the species being described in Dlabola (1979), where in fact it was not mentioned. On this basis the two earlier citations of the name must be nomina nuda. But the new combination given as *Iranodus repandus* was 2 years before the species was described. Accordingly we validate the name here in the combination *Iranodus repandus* (Dlabola 1981).

***Iranodus transversalis* Dlabola, 1980**

*Iranodus transversalis* Dlabola, 1980b: 204; Mirzayans (1995)

Iran localities: Bāft, Lalezār.

***Mycterodus***

This large issid genus is now often treated species placed in subgenera such as *Aconosimus*. But not all *Mycterodus* species have yet been placed in subgenera, so those species found in Iran are listed under *Mycterodus* only.

***Mycterodus astragalicus* Dlabola, 1974**

*Mycterodus astragalicus* Dlabola, 1974a: 42; Behdad (1988); Abaii (2000).

*Aconosimus astragalicus*; Dlabola (1997).

Iran localities: Kuhrang.

***Mycterodus demavendinus* Dlabola, 1981**

*Mycterodus demavendinus* Dlabola, 1981: 174; Behdad (1988), Abaii (2000).

*Aconosimus demavendinus*; Dlabola (1997).

Iran localities: Damāvand, Tochāl Mountain.

***Mycterodus elbursicus* (Logvinenko, 1974)**

*Mycterodus elbursicus* Logvinenko, 1974: 838

*Aconosimus elbursicus*; Dlabola (1983b), Dlabola (1997).

Iran localities: Shāhkuh-e Pāiin.

***Mycterodus fagetophilus* Dlabola, 1980**

*Mycterodus fagetophilus* Dlabola, 1980a: 62, Dlabola (1997).

Iran localities: Chālus.

***Mycterodus guilanicus* Dlabola, 1981**

*Mycterodus guilanicus* Dlabola, 1981: 176, Dlabola (1997).

Iran localities: Lāhijān.

***Mycterodus hezarmeshedi* Dlabola, 1980**

*Mycterodus hezarmeshedi* Dlabola, 1980a: 66

*Aconosimus hezarmeshedi*; Dlabola (1997).

Iran localities: Kuh-e Hezār.

***Mycterodus inassuetus* Dlabola, 1981**

*Mycterodus inassuetus* Dlabola, 1981: 176

*Aconosimus inassuetus*; Dlabola (1997).

Iran localities: Gazanak.

***Mycterodus kandavanicus* Dlabola, 1980**

*Mycterodus kandavanicus* Dlabola, 1980a: 63, Dlabola (1997).

Iran localities: Ghazvin, Kandovān (Māzandarān).

***Mycterodus krameri* Dlabola, 1974**

*Mycterodus krameri* Dlabola, 1974b: 297; Logvinenko (1974), Dlabola (1981), Dlabola (1997) [listed], Abaii (2000), Gnezdilov et al. (2004), Lashkari et al. (2009).

Iran localities: Behshahr, Bojnurd, Dasht, Gorgān, Rostamābād.

***Mycterodus lanceatus* Dlabola 1997**

*Aconosimus lanceatus* Dlabola 1997: 307

*Mycterodus lanceatus* Dlabola 1991, nomen nudum [The species appears not to be formally described in 1991].

Iran localities: Kelārdasht, Rudbār.

***Mycterodus peterseni* Dlabola, 1980**

*Mycterodus peterseni* Dlabola, 1980a: 68, Dlabola (1997)

Iran localities: Kandovān (Māzandarān).

***Mycterodus sexpunctatus* Dlabola, 1980**

*Mycterodus sexpunctatus* Dlabola, 1980a: 67; Gnezdilov et al. (2004).

*Aconosimus sexpunctatus*; Dlabola (1997).

Iran localities: Azādbar, Kandovān (Māzandarān).

***Mycterodus shahrudicus* Dlabola, 1980**

*Mycterodus shahrudicus* Dlabola, 1980a: 65

*Aconosimus shahrudicus*; Dlabola (1997).

Iran localities: Ghazvin.

***Pentissus bamicus* Dlabola, 1980**

*Pentissus bamicus* Dlabola, 1980b: 207; Mirzayans (1995).

Iran localities: Dehbakri, Dehpābid, Jiroft, Khutanābād, Taftān, Tamandān.

***Phasmena adyoungi* Dlabola, 1982**

*Phasmena adyoungi* Dlabola, 1982a: 131

Iran localities: Ghasr-e Ghand.

***Phasmena nasuta* Melichar, 1902**

*Phasmena nasuta* Melichar, 1902a: 93; Melichar (1906), Nast (1972) [listed], Dlabola (1981) [listed].

Iran localities: Temin.

Extralimital distribution: Turkey.

***Phasmena telifera* Melichar, 1902**

*Phasmena telifera* Melichar, 1902a: 92; Melichar (1906), Nast (1972) [listed], Dlabola (1981) [listed], Mirzayans (1995).

Iran localities: Sekand, Temin.

***Quadriva aurita* (Dlabola, 1982)**

*Hysterodus auritus* Dlabola, 1982a: 162

*Quadriva aurita* (Dlabola, 1982a); comb. n. in Gnezdilov et al. (2004).

Iran localities: Yāsuj.

***Quadriva dehbakrina* (Dlabola, 1980)**

*Hysterodus dehbakrinus* Dlabola, 1980b: 196

*Quadriva dehbakrina*; comb. n. in Gnezdilov et al. (2004).

Iran localities: Dehbakri.

***Quadriva lassa* (Dlabola, 1981)**

*Hysteropterum lassum* Dlabola, 1981: 178

*Quadriva lassa* (Dlabola, 1981): comb. n. in Gnezdilov et al. 2004.

Iran localities: Khānehkhoreh, Marg-e Malek, Shirāz.

***Quadriva ochaninei* (Puton, 1890)**

*Conosimus ochaninei* Puton, 1890: 232

*Conosimus oshanini*; Nast (1972) [listed].

*Quadriva ochaninei*; comb. n. in Gnezdilov (2010b).

Iran localities: Unknown locality.

Extralimital distribution: Central Asia.

Comment: Although the species had been listed in Nast (1972) from Iran, according to Gnezdilov (2010b) the species has only been collected from Central Asia.

***Quadriva proxima* (Dlabola, 1980)**

*Hysterodus proximus* Dlabola, 1980b: 198

*Quadriva proxima*; comb. n. in Gnezdilov et al. (2004).

Iran localities: Dehbakri.

***Quadriva sabzevarana* (Dlabola, 1980)**

*Hysterodus sabzevaranus* Dlabola, 1980b: 193

*Quadriva sabzevarana*; comb. n. in Gnezdilov et al. (2004).

Iran localities: Jiroft.

***Quadriva taftanica* (Dlabola, 1980)**

*Hysterodus taftanicus* Dlabola, 1980b: 197

*Quadriva taftanica*; comb. n. in Gnezdilov et al. (2004).

Iran localities: Taftān.

***Quadriva tangesarhena* (Dlabola, 1980)**

*Hysterodus tangesarhenus* Dlabola, 1980b: 193

*Hysterodus tangesarhensis*; Mirzayans (1995).

*Quadriva tangesarhena*; comb. n. in Gnezdilov et al. (2004).

Iran localities: Semirom, Sepidān, Tang-e Sarheh.

***Scorlupaster asiaticum* (Lethierry, 1878)**

*Hysteropterum asiaticum* Lethierry, 1878: 27; Dlabola (1981).

*Hysteropterum tshurtshurnum* Linnavuori 1957: 96

*Scorlupaster asiaticum*; comb. n. in Emeljanov (1972b).

Iran localities: Shirāz.

Extralimital distribution: Syria, Transcaucasia, Afghanistan, Central and Northern Asia.

***Scorlupaster emersum* (Dlabola, 1981)**

*Hysteropterum emersum* Dlabola, 1981: 184

*Scorlupella emersum*: Mirzayans (1995).

Iran localities: Robāt-e gharebil.

***Scorlupella montana* (Becker, 1865)**

*Issus montana* Becker, 1865

*Scorlupella montana*; Mozaffarian and Gnezdilov (in press).

Iran localities: Ālmeh, Dasht, Kandovān (Āzarbāijān-e Sharghi), Matnagh, Tāzekand, Yāichi.

Extralimital distribution: South Europe, Caucasus, Central Asia, Turkey, East Mediterranean (except North Africa).

***Tautoprosopa transcaspia* (Emeljanov, 1978)**

*Brachyprosopa transcaspia* Emeljanov, 1978: 332

*Verticisium pictifrons* (Melichar, 1906), sensu Dlabola (1979c), misidentification (see Gnezdilov 2002).

*Tautoprosopa transcaspia*; Gnezdilov (2002), Gnezdilov et al. (2004).

Iran localities: Dareh gaz, Khalkānlu, Khargh, Māzandarān, Shurlukh.

Extralimital distribution: Kazakhstan, Turkmenia (Gnezdilov 2002).

**Family Kinnaridae**

***Perloma boroumandi* (Dlabola, 1981)**

*Adolenda boroumandi* Dlabola, 1981: 138; Mirzayans (1995).

*Perloma boroumandi;* Emeljanov (1984).

Iran localities: Bāhukalāt, Geno, Ghasr-e Ghand, Nikshahr.

Comment: Listed as Cixiidae in Dlabola (1981).

***Perloma brunnescens* (Emeljanov, 1984)**

*Propleroma brunnescens*Emeljanov, 1984: 471

*Perloma brunnescens*; Wilson 2010a.

Iran localities: Kāravāndar.

Extralimital distribution: UAE (Wilson, 2010a).

***Perloma satrapa* (Dlabola, 1981)**

*Adolenda satrapa* Dlabola, 1981: 140

*Perloma satrapa*; Emeljanov (1984)*.*

Iran localities: Ghasr-e Ghand, Kāzerun.

Comment: Listed as Cixiidae in Dlabola (1981).

***Perloma zarudnyi* (Emeljanov, 1984)**

*Propleroma zarudnyi* Emeljanov, 1984: 474

*Perloma zarudnyi*; Wilson (2010a).

Iran localities: Kāravāndar.

**Family Lophopidae**

***Lophops pallidus* Melichar, 1902**

*Lophops pallidus* Melichar, 1902a: 90

*Lophops pallida*; Nast (1972) [listed], Dlabola (1981) [listed].

Iran localities: Anārak.

Extralimital distribution: Saudi Arabia (Dlabola, 1980c).

**Family Meenoplidae**

***Anigrus farsicus* Dlabola, 1986**

*Anigrus farsicus* Dlabola, 1986: 171

Iran localities: Borāzjān, Rāsk.

***Meenoplus albosignatus* Fieber, 1866**

*Meenoplus albosignatus* Fieber, 1866: 510; Dlabola (1984), Mirzayans (1995).

Iran localities: Sepidān.

Extralimital distribution: Southern Europe, Caucasus, North parts of Western Asia.

***Nisia nervosa* (Motschulsky, 1863)**

*Meenoplus atrovenosus* Lethierry, 1888: 466

*Nisia nervosa*; Mirzayans (1995).

Iran localities: Isin.

Extralimital distribution: widely distributed from subtropical and tropical regions of Old World. North of Africa, North parts of Western Asia, UAE and Eastern Asia.

**Family Nogodinidae**

***Hadjia nerii* Dlabola, 1981**

*Hadjia nerii* Dlabola 1981: 197

Iran localities: Isin.

***Hadjia quadrifasciata* Dlabola, 1981**

*Hadjia quadrifasciata* Dlabola 1981: 195

Iran localities: Doborji, Geno, Ghotbābād, Hājiābād.

***Iranissus ephedrinus* Dlabola, 1980, new placement**

*Iranissus ephedrinus* Dlabola, 1980b: 201

Iran localities: Bānuchārehar, Ferdows-e Esfandaghe, Geno, Hājiābād, Jiroft, Mohammadābād, Tis.

Comment: This species was described and placed in Issidae, We here transfer the species to Nogodinidae. (Suggested transfer by V. Gnezdilov pers. comm. and confirmed by MRW by examination of specimens).

***Morsina persica* Melichar, 1902**

*Morsina persica* Melichar, 1902a: 98; Nast (1972) [listed], Dlabola (1981), Mirzayans (1995).

Iran localities: Bazmān, Geno, Gorgān, Jāsk, Jiroft, Mumān, Sendarak.

***Philbyella glarea* Dlabola & Heller, 1962**

*Philbyella glarea* Dlabola & Heller, 1962: 2; Dlabola (1981), Mirzayans (1995), Nast (1972) [listed].

Iran localities: Bandar Abbās, Chābahār, Fāriāb, Kahnuj, Sirjān, Tang-e Sarheh.

**Family Ricaniidae**

***Pochazia umbrata* Melichar, 1896**

*Pochazia umbrata* Melichar, 1896: 385; Nast (1972) [listed].

Iran localities: Unknown locality.

Extralimital distribution: Turkmenia, Russia (Siberia) (Nast, 1972).

***Ricania hedenborgi* Stål, 1865**

*Ricania hedenborgi* Stål, 1865: 162; Mirzayans et al. (1976), (Dlabola (1981, 1983a [listed], 1984), Mirzayans (1995), Haghshenas and Khajehali (2000)

Iran localities: Chahārmahāl- Bakhtiāri province, Kāzerun, Shirāz, Sarvestān, Aliābād (Jahrom), Hamadān, Asadābād, Izeh.

Extralimital distribution: Greece, Turkey, Armenia.

***Ricania soraya* Dlabola, 1983**

*Ricania soraya* Dlabola, 1983a: 93

Iran localities: Ghaderābād, Kāzerun, Masiri.

**Family Tettigometridae**

***Eurychila pantherina* (Horváth, 1891)**

*Tettigometra pantherina* Horváth, 1891: 81

*Eurychila pantherina*; (Dlabola (1981, 1984), Mirzayans (1995).

Iran localities: Meshkinshahr, Minushahr.

Extralimital distribution: Transcaucasus, Afghnistan and Central Asia.

***Mitricephalus macrocephalus* (Fieber, 1865)**

*Tettigometra macrocephalus* Fieber, 1865: 569

*Mitricephalus macrocephalus*; Dlabola (1981).

Iran localities: Kandovān (Māzandarān), Lajrān, Tochāl Mountain.

Extralimital distribution: Europe, Turkey, Afghanistan, Central and Northern Asia.

***Tettigometra angulata* Lindberg, 1948**

*Tettigometra angulata* Lindberg, 1948: 19; Dlabola (1972) [listed], Nast (1972) [listed], Mirzayans (1995).

Iran localities: Evin, Kāshān, Rafsanjān.

Extralimital distribution: Transcaucasia, Turkey, Israel, Afghanistan and Central Asia.

***Tettigometra costulata* Fieber, 1865**

*Tettigometra costulata* Fieber, 1865: 572; Melichar (1902a), (Dlabola (1971a, 1972, 1981), Nast (1972) [listed], Behdad (1988), Rajabi (1989), Mirzayans (1995), Abaii (2000).

Iran localities: Aliābād (Ghom), Bazmān, Evin, Gazanak, Geno, Ghom, Karaj, Kuhrang, Māku, Marand, Mohammadābād.

Extralimital distribution: North Africa, Southern Europe, Yugoslavia, Transcaucasia, Northern parts of Western Asia, Afghanistan, Central Asia.

***Tettigometra demavenda* Dlabola, 1981**

*Tettigometra demavenda* Dlabola, 1981: 170; Dlabola (1981).

Iran localities: Gazanak, Lajrān.

***Tettigometra depressa* Fieber, 1865**

*Tettigometra depressa* Fieber, 1865: 563; Nast (1972) [listed].

Iran localities: Unknown locality.

Extralimital distribution: North Africa, Southern, Western and Eastern Europe, North West of Asia.

***Tettigometra eremi* Lindberg, 1948**

*Tettigometra eremi* Lindberg, 1948: 27; Nast (1972) [listed], (Dlabola (1972, 1981), Mirzayans (1995).

Iran localities: Ābyek, Āmol, Eynvarzān, Gazanak, Kandovān (Māzandarān), Lajrān, Robāt-e gharebil.

Extralimital distribution: Ukraine, Transcaucasia, Turkey, Afghnistan and Central Asia.

***Tettigometra hexaspina* Kolenati, 1857**

*Tettigometra hexaspina* Kolenati 1857: 428; Dlabola (1984)

Iran localities: Kāzerun, Khoy.

***Tettigometra pseudovitellina* Mitjaev, 1971**

*Tettigometra pseudovitellina* Mitjaev, 1971: 75; Dlabola (1981)

Iran localities: Āb Āsk, Marand.

Extralimital distribution: Kazakhstan (Nast, 1972).

***Tettigometra sordida* Fieber, 1865**

*Tettigometra sordida* Fieber, 1865: 571; Nast (1972) [listed].

Iran localities: Unknown locality.

Extralimital distribution: Western and Eastern Europe.

***Tettigometra sororcula* Horváth, 1897**

*Tettigometra sororcula* Horváth 1897a: 90; Dlabola 1994 [listed].

Iran localities: unknown locality.

Extralimital distribution: Southern Europe.

***Tettigometra sulphurea* Mulsant & Rey, 1855**

*Tettigometra sulphurea* Mulsant & Rey, 1855: 209; Mirzayans et al*.* (1976), Dlabola (1981), Mirzayans (1995).

Iran localities: Ābyek, Dārān, Delijān, Evin, Gāvkoshak, Geno, Ghazvin, Ghom- Esfahān Rd, Kandovān (Āzarbāijān-e Sharghi), Karaj, Rudehen.

Extralimital distribution: Southern, Western and Eastern Europe, North parts of Western Asia, Afghanistan and Central Asia.

***Tettigometra varia* Fieber, 1865**

*Tettigometra varia* Fieber, 1865: 565; Dlabola (1981), Mirzayans (1995).

Iran localities: Ghom, Robāt-e gharebil.

Extralimital distribution: Eastern Europe, Jordan, Afghanistan and Central Asia.

***Tettigometra vitellina* Fieber, 1865**

*Tettigometra vitellina* Fieber, 1865: 566; Barkhordari et al. (1981) [listed], Dlabola (1981), Mirzayans (1995), Mirzayans et al. (1976), Nast (1972) [listed].

Iran localities: Damāvand, Dasht-e arjan, Evin, Firuzkuh, Gazanak, Ghazvin, Kandovān (Māzandarān), Karaj, Māku, Marand, Robāt-e Tork, Saādatshahr, Tehrān.

Extralimital distribution: Yugoslavia, Transcaucasia, Northern parts of Western Asia, Afghanistan and Central Asia.

**Family Tropiduchidae**

***Kazerunia leguaniforma* Dlabola, 1977**

*Kazerunia leguaniforma* Dlabola, 1977a: 164; (Dlabola (1977a, 1981[listed]).

Iran localities: Chābahār.

***Kazerunia ochreata* Dlabola, 1974**

*Kazerunia ochreata* Dlabola, 1974a: 41; Dlabola (1977a).

Iran localities: Fasā, Kāzerun.

***Kazerunia undulata* Dlabola, 1977**

*Kazerunia undulata* Dlabola, 1977a: 166

Iran localities: Ghasr-e Ghand, Sarbāz.

***Ommatissus lybicus* Bergevin, 1930**

*Ommatissus binotatus* Fieber, 1876; Afshar (1937), Gardenhire (1959) [listed], Farahbakhsh (1961), Gharib (1998), Dlabola (1994), Mirzayans (1995), Abaii (2000).

*Ommatissus binotatus lybicus* Bergevin 1930: 20; Gharib (1966), (Behdad (1988, 1991).

Iran localities: Bāfgh Kahkom, Bam, Bandar Abbās, Geno, Isin, Jahrom, Jandagh, Khorramshahr, Khur va Biābānak, Mehrān, Shahdād, Tabas.

Comments: Asche and Wilson (1989) recognized the variety *lybicus* as a distinct species.

Extralimital distribution: Middle East (Asche and Wilson 1989), Saudi Arabia (Dlabola, 1979d), UAE (Wilson 2010b).

***Trypetimorpha fenestrata* Costa, 1862**

*Trypetimorpha fenestrata* Costa, 1862: 60; Dlabola (1981), Mirzayans (1995).

Specimens from Iran do not conform exactly to the male genitalia structure illustrated in Huang and Bourgoin (1993. Figs 40-47) but fall into the variation described for the species.

Iran localities: Dasht.

Extralimital distribution: Cyprus, Yugoslavia, Algeria, Israel, Italy (Huang and Bourgoin 1993), Kyrgizia (Novikov et al. 2006).

## List of the localities, their coordinates and the codes (Fig. 2)

Ābādeh 31°09'N, 52°39'E (F2)

Ābāsk 35°53'N, 52°10'E (M7)

Ābyek 36°02'N, 50°31'E (Gz2)

Ahram 28°53'N, 51°16'E (B4)

Ahvāz 31°19'N, 48°40'E (Kh10)

Akbarābād 29°14'N, 52°46'E (F11)

Akhlamad 36°36'N, 58°57'E (KhR4)

Albāji 31°29'N, 48°37'E (Kh8)

Aliābād (Ghom) 35°08'N, 50°59'E (Gh2)

Aliābād (Jahrom) 29°30'N, 52°35'E (F17)

Ālmeh 37°23'N, 56°8'E (Gs4)

Āmol 36°28'N, 52°21'E (M8)

Anārak 26°44'N, 62°15'E (S16)

Anbarābād 28°25'N, 57°50'E (Km10)

Anbarieh 32°19'N, 48°08'E (Kh1)

Andimeshk 32°27'N, 48°20'E (Kh1)

Asadābād 34°47'N, 48°07'E (Hd2)

Asālem 37°43'N, 48°54'E (G2)

Āzādbar 36°07'N, 51°10'E (T2)

Bābol 36°34'N, 52°42'E (M9)

Bābolsar 36°41'N, 52°38'E (M9)

Bāfgh 31°35'N, 55°24'E (Y4)

Bāft 29°14'N, 56°36'E (Km7)

Bāghak 28°54'N, 51°07'E (B4)

Bāhukalāt 25°43'N, 61°25'E (S18)

Bājgāh 29°43'N, 52°35'E (F9)

Bam 29°06'N, 58°21'E (Km11)

Bampur 27°11'N, 60°27'E (S9)

Bandar Abbās 27°11'N, 56°16'E (H7)

Bandar Anzali 37°28'N, 49°27'E (G4)

Bandar Chārak 26°43'N, 54°16'E (H15)

Bandar Khamir 26°56'N, 55°35'E (H5)

Bandar Lengeh 26°33'N, 54°52'E (H3)

Bānuchārehar 28°30'N, 57°00'E (Km8)

Bārgāh 27°38'N, 57°42'E (Km12)

Bazmān 27°49'N, 60°11'E (S5)

Behshahr 36°41'N, 53°32'E (M11)

Bidhand 33°14'N, 50°18'E (E2)

Bidruyeh 32°46'N, 48°13'E (Kh1)

Bidzard 29°21'N, 51°51'E (F6)

Bilāi 26°30'N, 57°06'E (H12)

Birjand 32°53'N, 59°12'E (KhJ1)

Bishāpur 29°43'N, 51°24'E (F5)

Bojnurd 37°28'N, 57°19'E (KhSh2)

Borāzjān 29°16'N; 51°12'E (B3)

Chābahār 25°18'N, 60°37'E (S19)

Chāhkuh 26°37'N, 54°58'E (H2)

Chālus 36°39'N, 51°25'E (M3)

Chenārshāhijān 29°51'N, 51°34'E (F5)

Dālaki 29°25'N, 51°17'E (B2)

Damāvand 35°44'N, 52°03'E (T7)

Dārāb 28°45'N, 54°32'E (F18)

Dārābād 35˚48'N, 51˚26'E (T4)

Dārān 32°58'N, 50°24'E (E6)

Dārbahāre 30°26'N, 49°54'E (Kh14)

Darband 36°01'N, 51°28'E (T4)

Dareh gaz 27°51'N, 56°17'E (H7)

Darpahn 26°44'N, 57°28'E (H12)

Darzin 27°58'N, 55°58'E (H6)

Dasht 37°17'N, 56°07'E (Gs4)

Dasht-e arjan 29°39'N, 51°58'E (F8)

Dasht-e Moghān (A)

Dehbakri 29°03'N, 57°55'E (Km9)

Dehbid 30°38'N, 53°13'E (F10)

Dehpābid 28°37'N, 60°46'E (S4)

Delijān 33°59'N, 50°40'E (Mz1)

Denā mt (west slpe) 30°49'N, 51°35'E (KB1)

Divāndareh 35°55'N, 47°01'E (Kd1)

Doborji 28°27'N, 55°06'E (F18)

Dowlatābād 28°20'N, 56°38'E (Km8)

Esfahān 32°40'N, 51°40'E (E3)

Esfarāyen 37°05'N, 57°29'E (KhSh4)

Eskandari 32°48'N, 50°25'E (E7)

Eslāmābād 37°42'N, 56°5'E (Gs5)

Espakeh 26°47'N, 60°14'E (S8)

Estahbān 29°07'N, 54°02'E (F16)

Evin 35°40'N, 51°26'E (T4)

Eynvarzān 35°39'N, 52°12'E (T8)

Fāriāb 27°28'N, 57°06'E (H10)

Fasā 28°55'N, 53°38'E (F15)

Feizābād 36°52'N, 54°33'E (Gs1)

Ferdows-e Esfadaghe 28°44'N, 57°15'E (Km10)

Firuzābād 28°50'N, 52°34'E (F13)

Firuzkuh 35°45'N, 52°45'E (T9)

Galugāh 36°43'N, 53°48'E (M11)

Gambuyeh 31°23'N, 48°30'E (Kh7)

Ganāveh 29°34'N, 50°31'E (B1)

Gāvbandi 27°12'N, 53°03'E (H1)

Gāvkoshak 29°38'N, 51°48'E (F7)

Gāvkoshi 28°38'N, 57°12'E (Km10)

Gazanak 35°54'N, 52°12'E (M6)

Geno 27°24'N, 56°12'E (H6)

Ghaderābād 30°20'N, 53°21'E (F2)

Ghāemshahr 36°28'N, 52°53'E (M9)

Gharechaman 37°36'N, 47°08'E (Ash4)

Ghāsemābād 27°10'N, 60°20'E (S8)

Ghasr-e Ghand 26°12'N, 60°45'E (S13)

Ghazvin 36°16'N, 50°00'E (Gz1)

Ghezel-Bolāgh 39°22'N, 44°12'E (AG1)

Gholhak 35°46'N, 51°26'E (T4)

Ghom 34°38'N, 50°53'E (Gh1)

Ghom Lake (Gh1)

Ghotbābād 27°46'N, 56°06'E (H6)

Golbāf 29°51'N, 57°44'E (Km5)

Gorgān 36°50'N, 54°25'E (Gs2)

Hafttappeh 32°02'N, 48°23'E (Kh2)

Hājiābād 28°08'N, 55°52'E (H6)

Hājiābād 28°18'N, 55°54'E (F18)

Hamadān 34°47'N, 48°30'E (Hd1)

Hamidieh 31°28'N, 48°26'E (Kh6)

Hāresābād 36°07'N, 57°36'E (KhR2)

Hasanlangi 27°21'N, 56°52'E (H9)

Hashtpar 37°48'N, 48°54'E (G1)

Heirān 38°22'N, 48°34'E (A2)

Hessār 36°02'N, 59°20'E (KhR6)

Hezār mt (Peak) 29°31'N, 57°15'E (Km6)

Hichān 26°20'N, 60°03'E (S11)

Hoseinābād 28°51'N, 54°14'E (F18)

Irānshahr 27°12'N, 60°41'E (S9)

Isin 27°19'N, 56°17'E (H7)

Izadkhāst 31°30'N, 52°07'E (F1)

Izeh 31°49'N, 49°51'E (Kh4)

Jahānābād 29°42'N, 53°51'E (F14)

Jahrom 28°30'N, 53°33'E (F17)

Jandagh 34°03'N, 54°24'E (E4)

Jāsk 25°38'N, 57°46'E (H14)

Jiroft 28°40'N, 57°43'E (Km10)

Kāhe 35°06'N, 58°09'E (KhR8)

Kahkom 28°12'N, 55°46'E (Y3)

Kahnuj 27°33'N, 57°42'E (Km12)

Kahurak 29°26'N, 59°39'E (S3)

Kahurestān 27°12'N, 55°34'E (H4)

Kamālābd 29°17'N, 53°02'E (F12)

Kamandān 33°18'N, 49°27'E (L2)

Kāmyārān 34°47'N, 46°56'E (Kd3)

Kandovān (Āzarbāijān-e Sharghi) 37°54'N, 46°05'E (Ash2)

Kandovān (Māzandarān) 36°08'N, 51°17﻿﻿'E (M6)

Kangān 27°50'N, 52°04'E (B6)

Karaj 35°49'N, 50°58'E (T2)

Kāravāndar 27°57'N, 60°43'E (S4)

Kāshān, 33°59'N, 51°26'E (E1)

Kāzerun 29°36'N, 51°39'E (F6)

Kelārdasht 32°32'N, 50°20'E (M5)

Kermān 30°14'N, 56°58'E (Km3)

Kermānshāh (Yazd province) 31°20'N, 54°54'E (Y2)

Khalkānlu 37°05'N, 58°44'E (KhR1)

Khalkhāl 37°37'N, 48°31'E (A3)

Khānehkhoreh 30°51'N, 53°08'E (F2)

Khargh 36°57'N, 58°00'E (KhSh3)

Kharposht 32°49'N, 59°02'E (KhJ1)

Khātunābād 29°28'N, 57°48'E (Km9)

Khorramābād 36°45'N, 50°53'E (M2)

Khorramshahr 30°25'N, 48°11'E (Kh12)

Khosro- Shirin 30°53'N, 52°00'E (F2)

Khoy 38°21'N, 44°50'E (AG5)

Khur va Biābānak 33°46'N, 55°05'E (E5)

Komehr 30°26'N, 51°52'E (F3)

Konārdān 28°37'N, 53°20'E (F17)

Kuhenjān 29°19'N, 52°54'E (F11)

Kuhrang 32°32'N, 50°20'E (Ch3)

Kushk 30°31'N, 51°30'E (F4)

Lāhijān 37°12'N, 50°00'E (G5)

Lajrān 35°16'N, 52°08'E (T7)

Lalezār 29°31'N, 56°48'E (Km6)

Leshtar 32°02'N, 50°16'E (Ch1)

Māhān 30°05'N, 58°18'E (Km5)

Mahārlu 29°21'N, 52°49'E (F11)

Māku 39°17'N, 44°31'E (AG2)

Malārd 35°40'N, 50°58'E (T3)

Marāgheh 37°22'N, 46°14'E (Ash6)

Marand 38°25'N, 45°46'E (Ash1)

Marg-e-Malek 32°29'N, 50°29'E (Ch1)

Mashhad 36°17'N, 59°35'E (KhR5)

Mashhad-e Ardehāl 34°1'N, 51°2'E (E1)

Masiri 30°15'N, 51°30'E (F4)

Matnagh 37°50'N, 46°31'E (Ash2)

Mehrān 33°06'N, 46°09'E (I1)

Meshkinshahr 38°23'56"N; 47°40'55"E (A1)

Miāneh 37°25'N, 47°42'E (Ash7)

Miānjangal 29°06'N, 53°42'E (F15)

Mijān 28°42'N, 57°56'E (Km10)

Mināb 27°08'N, 57°05'E (H11)

Minushahr 30°20'N, 48°13'E (Kh13)

Mohammadābād 28°57'N, 57°55'E (Km9)

Mollāsāni 31°35'N, 48°52'E (Kh9)

Mumān 25°35'N, 60°29'E (S19)

Nagsh-e Rostam 29°59'N, 52°54'E (F10)

Nāhārkhorān 36°45'N, 54°27'E (Gs2)

Nehbandān 31°41'N, 60°02'E (KhJ2)

Nikshahr 26°13'N, 60°13'E (S12)

Nishābur 36°03'N, 59°06'E (KhR3)

Nosratābad 29°03'N, 57°56'E (Km8)

Nowshahr 36°38'N, 51°30'E (M4)

Nugh 34°17'N, 58°09'E (KhR8)

Nurābād 30°06'N, 51°31'E (F4)

Orumieh 37°33'N, 45°4'E (AG4)

Orumieh lake (AG)

Pākuh 27°55'N, 61°30'E (S6)

Parehsar 37°37'N, 49°02'E (G3)

Paskuh 27°33'N, 61°38'E (S6)

Pishin 26°04'N, 61°25'E (S17)

Pol-e Tang 32°51'N, 48°02'E (L1)

Posht- e kuh 29°25'N, 56°50'E (Km6)

Rafsanjān 30°24'N, 55°58'E (Km1)

Rāmiān 37°01'N, 55°08'E (Gs1)

Rāsk 26°14'N, 61°23'E (S15)

Rek 32°15'N, 59°7'E (KhJ1)

Rezvāndeh 37°33'N, 49°09'E (G3)

Robāt-e gharebil 37°21'N, 56°16'E (KhSh1)

Robāt-e Tork 33°44'N, 50°50'E (Mz2)

Rostamābād 36°54'N, 49°27'E (G7)

Rudān 27°32'N, 57°25'E (H10)

Rudbār 36°48'N, 49°23'E (G7)

Rudbārak 36°04'N, 52°32'E (M5)

Rudehen 35°44'N, 51°51'E (T6)

Saādatshahr 30°06'N, 53°07'E (F10)

Sabzevār 36°12'N, 57°40'E (KhR2)

Sanandaj 35°19'N, 46°59'E (Kd2)

Sangān 28°35'N, 61°19'E (S4)

Sangrud 36°40'N, 49°38'E (G7)

Sarāvān 27°08'N, 62°14'E (S7)

Sarbāz 26°39'N, 61°14'E (S14)

Sāri 36°33'N, 53°02'E (M9)

Sarvestān 29°16'N, 53°12'E (F12)

Sekand 26°43'N, 61°31'E (S14)

Semirom 31°24'N, 51°34'E (E8)

Sendarak 26°50'N, 57°25'E (H12)

Sepidān 29°58'N, 52°23'E (F3)

Shabestar 38°10'N, 45°41'E (Ash3)

Shādegān 30°39'N, 48°40'E (Kh11)

Shahdād 30°25'N, 57°42'E (Km4)

Shāhkuh-e Pāiin 36°34'N, 54°26'E (Gs3)

Shahr-e Kord 32°19'N, 50°51'E (Ch2)

Shāhrud 36°25'N, 55°01'E (Sm1)

Shemirān 35°49'N, 51°24'E (T4)

Shieh 35°05'N, 50°10'E (G6)

Shirāz 29°37'N, 52°31'E (F9)

Shul 30°02'N, 52°09'E (F9)

Shurlukh 36°20'N, 60°35'E (KhR7)

Shusf 32°00'N, 59°53'E (KhJ2)

Shush 32°11'N, 48°14'E (Kh2)

Shushtar 32°02'N, 48°51'E (Kh3)

Silvāneh 37°25'N, 44°51'E (AG3)

Sirik 26°30'N, 57°06'E (H13)

Sirjān 29°26'N, 55°40'E (Km2)

Sisakht 30°47'N, 51°33'E (KB1)

Sufiān 38°16'N, 45°58'E (Ash3)

Susangerd 31°33'N, 48°11'E (Kh5)

Suza 26°46'N, 56°03'E (H8)

Tabas 33°35'N, 56°54'E (Y1)

Tabriz 38°05'N, 46°17'E (Ash2)

Taftān 28°36'N, 61°07'E (S4)

Tākestān 36°03'N, 49°42'E (Gz2)

Tamandān 28°36'N, 61°04'E (S4)

Tang-e Sorkheh 26°29'N, 60°02'E (S11)

Tāzekand 38°16'N, 47°14'E (Ash5)

Tehrān 35°41'N, 51°24'E (T4)

Temin 28°40'N, 61°08'E (S4)

Tis 25°21'N, 60°37'E (S19)

Tochāl Mountain 35°53'N, 51°26'E (T4)

Tonekābon 36°49'N, 50°52'E (M1)

Varāmin 35°19'N, 51°39'E (T5)

Veresk 35°56'N, 52°58'E (M12)

Yāichi 37°35'N, 46°11'E (Ash2)

Yāsuj 30°39'N, 51°35'E (KB2)

Yazd 31°54'N, 54°20'E (Y2)

Zābol 31°02'N, 61°29'E (S1)

Zāboli 27°07'N, 61°40'E (S10)

Zāhedān 29°29'N, 60°51'E (S2)

Zanjān 36°40'N, 48°28'E (Z1)

Zarghān 29°46'N, 52°43'E (F10)

Zeidān 27°58'N, 51°31'E (B5)

Ziārān 36°06'N, 50°31'E (T1)

Ziārat 27°41'N, 57°05'E (H11)

Zirāb 36°10'N, 52°59'E (M10)

Zoshk 36°20'N, 59°10'E (KhR4)
